# *Xanthomonas* infection and ozone stress distinctly influence the microbial community structure and interactions in the pepper phyllosphere

**DOI:** 10.1038/s43705-023-00232-w

**Published:** 2023-03-27

**Authors:** Rishi Bhandari, Alvaro Sanz-Saez, Courtney P. Leisner, Neha Potnis

**Affiliations:** 1 Department of Entomology and Plant Pathology, Auburn University, Auburn, AL 36849 USA; 2 Department of Crop, Soil and Environmental Sciences, Auburn University, Auburn, AL 36849 USA; 3 Department of Biological Sciences, Auburn University, Auburn, AL 36849 USA

**Keywords:** Microbial ecology, Metagenomics

## Abstract

While the physiological and transcriptional response of the host to biotic and abiotic stresses have been intensely studied, little is known about the resilience of associated microbiomes and their contribution towards tolerance or response to these stresses. We evaluated the impact of elevated tropospheric ozone (O_3_), individually and in combination with *Xanthomonas perforans* infection, under open-top chamber field conditions on overall disease outcome on resistant and susceptible pepper cultivars, and their associated microbiome structure, function, and interaction network across the growing season. Pathogen infection resulted in a distinct microbial community structure and functions on the susceptible cultivar, while concurrent O_3_ stress did not further alter the community structure, and function. However, O_3_ stress exacerbated the disease severity on resistant cultivar. This altered diseased severity was accompanied by enhanced heterogeneity in associated *Xanthomonas* population counts, although no significant shift in overall microbiota density, microbial community structure, and function was evident. Microbial co-occurrence networks under simultaneous O_3_ stress and pathogen challenge indicated a shift in the most influential taxa and a less connected network, which may reflect the altered stability of interactions among community members. Increased disease severity on resistant cultivar may be explained by such altered microbial co-occurrence network, indicating the altered microbiome-associated prophylactic shield against pathogens under elevated O_3._ Our findings demonstrate that microbial communities respond distinctly to individual and simultaneous stressors, in this case, O_3_ stress and pathogen infection, and can play a significant role in predicting how plant-pathogen interactions would change in the face of climate change.

## Introduction

The phyllosphere (aboveground parts) of plants is a unique, nutrient-poor habitat and is inhabited by various prokaryotic and eukaryotic microorganisms [1] that colonize either the leaf surface (epiphytes) or inside the leaf tissue (endophytes) [2, 3]. Leaf microbial community assembly and succession are influenced by deterministic and stochastic processes. Although dispersal from neighboring plants and other demographic factors such as neighbor identity and age are contributing factors toward phyllosphere microbiome diversity [4], plant host factors such as host genotype, developmental stage [5], and host resistance [6] shape the microbiome assembly. This host filtering of the microbiome is observed due to different resource availability on the leaf surface [7], differing physical properties [8], and host defense signaling [9, 10].

Members of the phyllosphere microbiome are known to play a role in nutrient acquisition [11], plant growth and productivity [12] and tolerance to various biotic and abiotic stresses [13–17]. Pathogen invasion is one of the most influential biotic stresses affecting the plant microbial assembly in the phyllosphere [18]. Pathogens can modify the habitat by secretion of virulence factors, biosurfactants, or hormones, thereby increasing resource availability for other resident colonizers including opportunists to flourish [19, 20]. Pathogens can also influence resident microflora through niche or resource competition [1, 18, 19, 21]. Plant defense signaling activated in response to pathogen attack has also been indicated as a source of alteration of the phyllosphere community [16, 22]. Regardless of the source of change to the phyllosphere community, dominant members are thought to restore stability to this disturbed community [23]. Furthermore, increasing evidence has suggested that plants can recruit microbes in the phyllosphere that offer protection against pathogen [24–26], indicating disease-suppressive microbiome assembly in the phyllosphere in response to pathogen similar to what has been observed in the rhizosphere [27, 28]. Phyllosphere microbial community structure and composition is also shaped by host plant’s response to abiotic stresses, such as drought [29, 30], increase in surface temperature or warming [31–33], elevated CO_2_ [34], and ultraviolet radiation [35].

Abiotic stressors can alter host susceptibility to pathogens by interfering with defense hormone signaling [36] and thus influence disease incidence. Exposure of plants to simultaneous biotic and abiotic stressors can result in positive or negative impacts on plant responses depending on the timing, nature, and severity of each stress, as different defense signaling pathways may interact or inhibit each other [37, 38]. Furthermore, recent work has demonstrated that climate change may lead to increased incidence of disease outbreaks due to the spread of pathogens outside their geographical range [39]. Taken together, there are many internal and external factors that can shape the phyllosphere microbiome, and work is needed to fully understand the role that phyllosphere microbiome plays in the plant’s response to simultaneous biotic and abiotic stressors.

One such abiotic stressor that plants experience is elevated levels of tropospheric ozone (O_3_). Global warming caused by greenhouse gases has resulted in the increase of tropospheric O_3_ due to the rise in precursors such as nitrogen oxide (NOx), CO, methane, and other volatile organic compounds [40, 41]. A study across the US predicted that the 5–95^th^ percentile for daily 8-h maximum O_3_ will increase from 31–79 parts per billion (ppb) in 2012 to 30–87 ppb in 2050 [42]. This increase in O_3_ level is significant as O_3_ concentrations above 40 ppb are highly phytotoxic [43]. Elevated O_3_ can negatively impact plants and many levels, including visible injury and reduction in photosynthesis, which in turn affects plant growth, nutritional value, crop yield, and alterations to carbon allocation [43–45]. As we learn more about how climate change associated abiotic and biotic stressors influence plant response at the molecular, cellular or transcriptomic level, important questions to address are how associated microbiome would respond to or contribute to plant’s response in the presence of individual or simultaneous stressors and whether critical ecological functions of phyllosphere microbial communities would be altered in presence of stressors.

To address these questions, we explicitly focused on the response of the phyllosphere microbiome of two pepper cultivars differing in resistance towards a foliar pathogen, *Xanthomonas perforans*, in presence of ambient and elevated O_3_ levels. We used an experimental setup in the field involving open-top chambers (OTCs) that allowed us to manipulate O_3_ levels and dissect the influence of genotype x environment (G x E) interactions on the overall outcome of plant disease as well as on microbiome structure and function. The two pepper cultivars used in this study differed in their resistance against *X. perforans*, an emerging pepper pathogen in the southeastern US: one being susceptible cultivar Early Cal Wonder and the other being commercial cultivar PS 09979325, largely deployed in the southeastern US and known to have polygenic quantitative resistance against all eleven races of the bacterial spot pathogen [46]. This specific host-pathosystem allowed us to not only study the response of the resistant variety under combined stressors, thereby, assessing its durability under altered climatic conditions, but also to test the response of the emerging pepper pathogenic species, *X. perforans* [47], on the susceptible and commercial resistant varieties under an altered environment. We hypothesized that phyllosphere microbial communities will show alterations in both taxonomic and functional profiles and altered seasonal dynamics in response to altered O_3_ levels, regardless of the cultivars. Interestingly, the influence of elevated O_3_ on plant susceptibility depends on the lifestyle of the pathogen. Such differential effects could stem from physiological differences, pathogen biology or differences in defense signaling pathways [48–50]. We hypothesized that presence of elevated O_3_ will increase overall susceptibility of pepper to bacterial spot xanthomonads, even on the resistant cultivar. We also hypothesized that establishment of disease would disrupt seasonal dynamics of the phyllosphere microbiome, and this effect will be stronger in the environments that support high disease pressure. Our experimental design allowed us to address the influence of elevated O_3_ on the overall disease outcome on cultivars differing in their resistance towards pathogen as well as facilitated assessment of taxonomic and functional profiles of the phyllosphere microbiome under simultaneous stressors. Lastly, as studies have indicated the importance of functions rather than species in community structure and assembly [51], we compared functional profiles of microbiomes to see whether ecological functions of the community are rather conserved regardless of biotic or abiotic stressors.

## Materials and methods

### Experimental site and design

The experiment was conducted at the Atmospheric Deposition (AtDep) site at Auburn University (Fig. S1A) in the 2021 growing season (May–July), where we harnessed OTCs (Fig. S1B) that allowed us to test the effect of O_3_ stress on plant-pathogen-microbiome interactions and address the complexity of plant defense-development trade-off. We used 12 chambers for fumigation, where six chambers had an ambient environment, and six had elevated O_3_ (Fig. S1A). Each elevated O_3_ chamber contains four O_3_ generators (HVAC-1100 Ozone generator, Ozone Technologies, Hull, IA, USA), equipped with two ultraviolet bulbs (Model GPH380T5VH/HO/4 P, Ozone Technologies, Hull, IA, USA) to generate the O_3_. Generators and bulbs are located within the elevated O_3_ chamber fan boxes. To reach the desired set-point of O_3_ (~100 ppb), O_3_ generators were controlled by 0–10 V control wires, which are controlled via an analog output module. To fumigate the plants, the ozonated air was blown from the fan box into the plastic lining of the open-top chamber (Fig. S1B). The plastic panel on the lower portion of the chamber is double-walled with holes on the inside panel, allowing O_3_ to be released over the plants inside the chamber. Each chamber is connected via plastic tubing to a central gas manifold to which each chamber is opened sequentially by 3-way solenoid valves. A microcontroller cycles through the 12 solenoid valves every 24 min (sampling each of the 12 chambers for 2 min) to monitor O_3_ from each chamber (Model 205 Dual Beam Ozone Monitor, 2B Technologies, Boulder, CO, USA) during the fumigation window (10 am to 6 pm). During this experiment, the average [O_3_] in the control chambers was around 30.6 ppb, while the fumigated chambers had an average [O_3_] of about 90.3 ppb (Fig. S1C). O_3_ levels in the elevated chambers were significantly higher during the growing season when compared to the ambient chambers (Kruskal–Wallis*, p* = 0.04) while the O_3_ levels between the elevated chambers were similar (*p* = 0.62).

Inoculation was performed on 5–6 weeks old seedlings of both cultivars. Plants were inoculated with a *X. perforans* suspension adjusted to 10^6^ CFU/ml in MgSO_4_ buffer amended with 0.0045% (vol/vol) Silwet L-77 (PhytoTechnology Laboratories, Shawnee Mission, KS, USA). The control plants were dip-inoculated in MgSO_4_ buffer amended with 0.0045% (vol/vol) Silwet L-77 (Fig S1D). The dip-inoculated plants were transplanted into sterile 10-inch plastic pots (The HC Companies, OH) with soil-less potting medium (Premier Tech Horticulture, PA). The pots were then transferred to the above-mentioned OTCs and maintained inside the OTCs throughout the growing season until harvest. In each of the chambers, we had six plants, each of Early Cal Wonder (referred to hereafter as the susceptible cultivar) and PS 09979325 (referred hereafter as the resistant cultivar) (Fig. S1E). Among the 12 chambers, plants in 6 chambers (three ambient and three elevated O_3_) were inoculated with the pathogen *X. perforans* while 6 chambers (three ambient and three elevated O_3_) had control plants inoculated with MgSO_4_ buffer (Fig. S1A).

### Disease severity measurements

The overall disease development was evaluated by estimating the percentage of disease symptoms caused by bacterial spot after transforming the Horsfall-Barratt ratings [52] to the midpoint of the rating range during both the mid and end of the season [53].

### Sample collection, DNA extraction, sequencing, and quality trimming

Pepper leaf samples were collected from both inoculated and control samples of each cultivar separately after inoculation with *Xanthomonas* or MgSO_4_ buffer and before keeping the plants in the chambers (base samples), followed by two other time points during the growing season (mid and end of the season). For each timepoint, leaves from 6 plants of each cultivar grown inside one chamber were pooled, so we have one sample per cultivar. During sampling, leaves were collected randomly to avoid bias towards diseased leaves and with at least one leaf per plant for each cultivar. 40 grams of leaf samples were sonicated for 15 min in phosphate-buffered saline solution (50 mM) amended with 0.02% Tween 20 and the dislodged cells were pelleted down and processed for DNA extraction. Briefly, total DNA was extracted using Wizard® Genomic DNA Purification Kit (Promega, Madison, WI) as per manufacturer instructions with the addition of a phenol:chloroform:isoamyl alcohol (25:24:1) followed by ethanol precipitation. The DNA was quantified using a Qubit 3.0 fluorometer (Thermo Fischer, Waltham, MA) and the DNA samples were submitted to the Duke Center for Genomic and Computational Biology sequencing core (Duke University, Durham, NC) for library preparation, and paired-end reads (2 × 150 bp) were sequenced on NovaSeq 6000 S1 flow cell system. The raw reads were then trimmed for quality using BBDuk (http://jgi.doe.gov/data-and-tools/bb-tools/) followed by host contamination removal with KneadData (https://bitbucket.org/biobakery/kneaddata/) using pepper cv. 59 (GCA_021292125.1) genome as a reference.

### Taxonomic profiling

Quality controlled and host decontaminated reads were taxonomically assigned using Kraken2 (v2.1.2) [54] against a standard Kraken2 database containing RefSeq libraries [55] of archaeal, bacterial, human, and viral sequences (as of March 01, 2022). Kraken2 is a kmer based short read classification system that assigns a taxonomic identification to each sequencing read by using the lowest common ancestor (LCA) of matching genomes in the database and has been used for high accuracy classification of metagenomic reads [56, 57]. Kraken2 report files were used as inputs to run Bayesian re-estimation of abundance with the Kraken (Bracken) (v2.6.2) [58] to re-estimate abundance at each taxonomic rank for all the samples. Bracken uses the taxonomy labels assigned by Kraken to estimate the abundance of each species. The database for Bracken was subsequently built with the Kraken2 database using the default 35 k-mer length and 100 bp read lengths based on the average read length in our sample with the lowest read length to re-estimate the relative abundance of microbial communities at the species level. The outputs from Bracken were combined using the combine_bracken_outputs.py function for downstream analysis. The kraken-biom tool (https://github.com/smdabdoub/kraken-biom) was used to convert the output from Bracken into BIOM format tables for diversity analyses in R [59].

In addition to relative abundance for each taxon, we calculated an estimate of absolute abundance based on relative abundance of different bacterial taxa and the total DNA recovered from each sample. Microbiota density described as total DNA (ng) per mg of fresh sample was calculated for each sample, which was then used to calculate the absolute abundance of different microbial taxa as defined by ng of DNA per mg of sample multiplied with the relative abundance [60].

The taxonomic composition and diversity of eukaryotes in the samples were accessed using the EukDetect (v1.3) [61]. EukDetect aligns the metagenomic reads to universal marker genes from conserved gene families curated from fungi, protists, non-vertebrate metazoan, and non-streptophyte archaeplastida genomes and transcriptomes followed by low-quality and duplicate reads filtering. The final eukaryotes abundance is calculated by filtering taxa with fewer than four reads and aligning to less than two marker genes. The resulting absolute abundance (Reads Per Kilobase of Sequence) was used to compare the diversity across the samples. The RPKS value was normalized by multiplying with a scaling factor calculated by dividing the median library size by the sample library size, which was then used to compare across the samples.

### Culture-dependent method for determining the *Xanthomonas* population

To determine the effect of cultivar and environment on the abundance of *X. perforans*, a culture-dependent method was used for tracking the *in planta* population of *Xanthomonas*. Plants (6 from each cultivar/chamber) were dip-inoculated as described earlier and kept inside the chambers with ambient and elevated O_3_. Leaf samples were taken at day 0, 7 and 14 after inoculation to determine the *in planta* bacterial population. At each sampling time, approximately 4 cm^2^ of leaf area was taken using a sterile cork borer and was macerated using a sterile Dremel® in microcentrifuge tubes with 1 ml of sterile 0.01 M MgSO_4_ buffer. The homogenized suspension was then diluted by ten-fold followed by plating on Nutrient Agar plate using a spiral plater (Neu-tecGroup Inc., NY). Plates were then incubated at 28 °C for 3 days and bacterial population was determined as colony forming units per centimeter squared of leaf area.

### Diversity, statistical analysis, and network analysis

All statistical and diversity analyses were performed using R (v4.1.3) [59] and Rstudio [62] with the *Phyloseq* (v1.38.0) [63], *vegan* (v2.5–7) [64], and *ggplot2* (v3.3.5) [65] packages. Before data analysis, the library size was normalized using scaling with ranked subsampling with ‘SRS’-function in the *SRS* R package (v0.2.2) [66]. Alpha diversity measures Chao1 and Shannon index were used to identify community richness and diversity, respectively. The Wilcoxon rank sum test tested significant differences in alpha diversity indices for nonparametric data and the T-test for normally distributed data. The appropriateness of these methods was verified by checking for the normal distribution of residuals based on the Shapiro–Wilk test for normality.

The differences in overall microbial profiles among the cultivars and different environmental conditions (β-diversity) were estimated using the Bray–Curtis distance. To understand the factors contributing to microbial community structure, we performed permutation multivariate analysis of variance (PERMANOVA) [67] as implemented in the adonis2 (analysis of variance using distance matrices, ADONIS) with the argument ‘by’ set to ‘margins’ and analysis of similarities (ANOSIM) with 1000 permutations (*p* = 0.05) using Bray–Curtis dissimilarity in the *vegan* R package (v 2.5–7). In addition, multivariate homogeneity of group dispersion test (BETADISPER) [68] was performed to determine the homogenous dispersion between the factors in relation to their microbial taxa. Non-metric multidimensional scaling (NMDS) among the sample groups was calculated using Bray–Curtis dissimilarity and visualized using the *ggplot2* package in R.

For our network analysis, the taxonomic data was subsetted to at least 0.5% relative abundance in over 20% of the samples (prevalence) to ensure that all samples had sufficient sequencing depth to recover most of the diversity. Correlation network analysis was performed using the SPRING [69] approach implemented in the R package *NetCoMi* (v1.1.0) [70]. Community structures across the treatment were estimated using the “cluster_fast_greedy” algorithm [71], and hub taxa were determined using the threshold of 0.95. A Jaccard index was used to test for similarities (Jacc = 0, lowest similarity and Jacc = 1, highest similarity) in selected local network centrality measures (degree, betweenness centrality, closeness centrality, and eigenvector centrality) to determine the hub or keystone taxa. A quantitative network assessment was performed with a permutation approach (1000 bootstraps) with an adaptive Benjamini–Hochberg correction for multiple testing.

### Functional profiling

Functional profiling of the microbial communities was conducted on concatenated paired-end sequences with HUMAnN3 (v3.0) [72] to quantify gene abundance (UniRef90 gene-families) [73] and MetaCyc pathways [74]. ChocoPhlAn nucleotide database v30 was used for functional pathway abundance and coverage estimation. The gene families and pathway abundance tables were sum-normalized to copies per million reads (CPM) to facilitate comparisons between samples with different sequencing depths. The output from HUMAnN3 was then imported into QIIME2 (v2021.11) [75] to generate nonmetric multidimensional scaling (NMDS) ordinations using Bray–Curtis dissimilarly matrix. To understand the factors driving functional profiles, we performed permutation multivariate analysis of variance (PERMANOVA) [67] as implemented in the adonis2 (analysis of variance using distance matrices, ADONIS) and analysis of similarities (ANOSIM) with 1000 permutations (*p* = 0.05) with different factors (cultivars, environment, inoculation status, and time of sampling), as described above. Differentially abundant pathways across the treatment were identified using the LEfSe (Linear discriminant analysis Effect Size) (v1.1.2) [76]. Pathways with a corrected *p* value of 0.05 or less and Linear Discriminant Analysis (LDA) score of log >2.5 were classified as significantly increased within one of the two groups.

## Results

### Influence of O_3_ levels on disease severity on resistant and susceptible cultivars

**Fig. 1:**
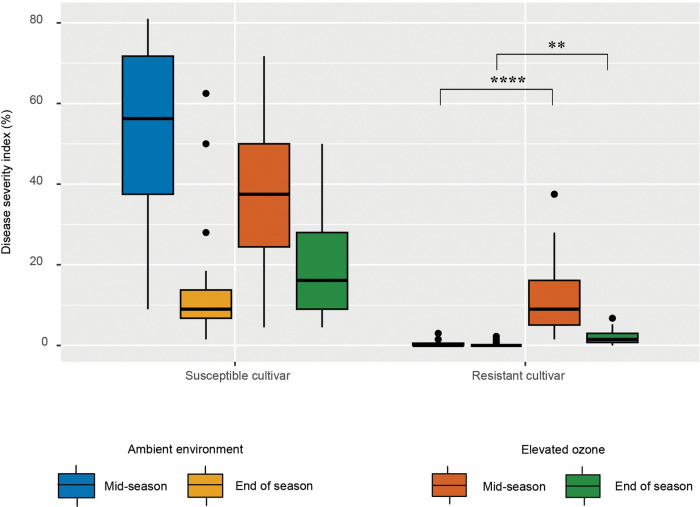
Elevated O_3_ exacerbates bacterial spot disease severity on the resistant cultivar but has no effect on the susceptible cultivar. Box and whisker plots showing the disease severity index (represented as % value) under elevated O_3_ and ambient environmental conditions across susceptible and resistant cultivars. Significance levels for each of the treatment combination are indicated by *p < 0.05; **p < 0.01; ***p < 0.001; ****p < 0.0001.

Overall higher disease severity index was recorded on the susceptible cultivar compared to the resistant cultivar. Under ambient conditions, the susceptible cultivar supported an average of 53.01% disease severity index during mid-season, which decreased to 15.11% by the end of the growing season. The resistant cultivar supported minimal disease with disease severity index of 0.37% during mid-season and 0.29% by the end of the season. Elevated O_3_ did not impact disease severity on the susceptible cultivar. However, significantly higher disease severity index was observed on the resistant cultivar under elevated O_3_ conditions, both at mid-season (12.61%) (*p* < 0.001) and end of the season (2.01%) (*p* = 0.01) compared to the ambient environment (mid-season = 0.37%, end of the season = 0.29%) (Fig. 1, Table S1).

### Sequencing statistics

The samples collected in the beginning of the experiment (base samples) and twice during the growing season (mid-season and end of the season) were subjected to shotgun metagenome sequencing, which produced 2.83 to 17.16 Gbps of raw reads per sample. Adapter trimming and removal of low-quality reads resulted in the loss of 4.3 to 11.3% of the total reads among different samples. Of the quality trimmed reads, 5.78 to 39.09% of the reads were identified as host reads and removed from further analysis. The samples at the early seedling stage yielded very few reads upon filtering because of higher host contamination (23–39%), indicating minimal microbial colonization in the greenhouse-grown seedlings before transplanting. Around 50.61% to 84.56% of the original total reads were retained for downstream analysis (Table S2).

### Microbial diversity and richness are reduced under susceptible response, but are not significantly affected by elevated O_3_

**Fig. 2:**
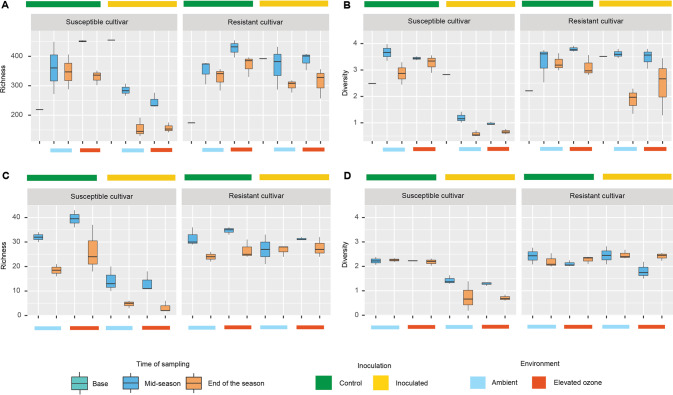
Elevated O_3_ has little impact on microbial diversity and richness. However, pathogen infection on susceptible cultivar reduces microbial community richness and diversity. **A** Bacterial Chao1 richness and **B** bacterial Shannon diversity index across different environments. **C** Eukaryotic community diversity and **D** richness across different treatments. Inoculated and control samples are indicated with yellow and green bars on the top, while ambient and elevated O_3_ treatments are denoted by light blue and red color bars at the bottom, respectively.

We next investigated the effect of inoculation and elevated O_3_ and their interaction on overall microbial diversity and richness of the phyllosphere communities. Overall bacterial richness and diversity values in both the mid and end of the season samples were higher in control plants when compared with base samples. This could be attributed to low microbial colonization levels on greenhouse-grown base samples that increased in diversity and richness upon exposure to natural field conditions. Eukaryotic diversity in the base samples was not calculated as these samples had reads counts below the threshold (fewer than 4 reads that align to fewer than 2 marker genes) to be considered present in the sample. The O_3_ stress alone did not influence bacterial (Table S3) and eukaryotic richness and diversity (Table S4) in both cultivars. However, pathogen infection led to significantly lower bacterial richness (*p* < 0.001) (Fig. 2A) and diversity (Kruskal–Wallis*, p* = 0.01) (Fig. 2B) as well as lower eukaryotic richness (Kruskal–Wallis, *p* = 0.01) (Fig. 2C) and diversity (Kruskal–Wallis, *p* = 0.02) (Fig. 2D) on the susceptible cultivar under ambient conditions throughout the growing season compared to that on control plants. Under combined stress of pathogen and elevated O_3_, there was a significant effect on both richness (*p* = 0.01) and diversity (*p* = 0.04) only during end of the season on the susceptible cultivar. Inoculation and elevated O_3_ did not influence bacterial richness (*pinoc* = 0.81, *penv* = 0.07) (Fig. 2A) and diversity (*pinoc* = 0.27, *penv* = 0.62) (Fig. 2B), or eukaryotic richness (Kruskal–Wallis, *pinoc* = 0.08, penv = 0.31) (Fig. 2C) and diversity (Kruskal–Wallis, *pinoc* = 0.23, *penv* = 0.82) (Fig. 2D) on the resistant cultivar. Time of sampling had significant influence on bacterial richness and diversity (*p* < 0.01) in both the cultivars.

### The effect of O_3_ levels was significant on the eukaryotic community, yet was minimal in shaping bacterial community structure

**Fig. 3:**
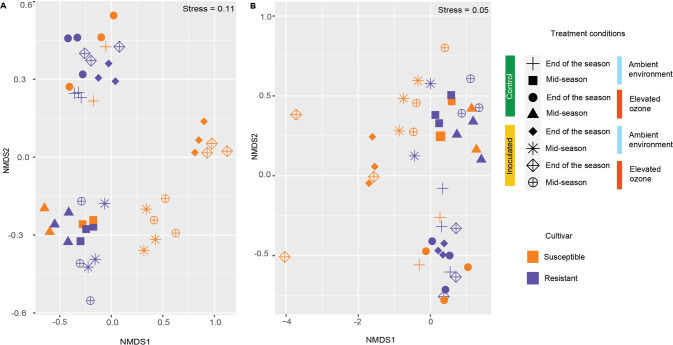
Elevated O_3_ changes microbial community structure on susceptible cultivars challenged with pathogen infection, but not on resistant cultivars. **A** Nonmetric Multidimensional Scaling (NMDS) ordination comparing the bacterial community diversity across two cultivars, environmental conditions, and time of sampling. **B** NMDS ordination comparing the eukaryotic community diversity across two cultivars, environmental conditions, and time of sampling.

To visualize the differences in bacterial and eukaryotic community structure between samples from two pepper cultivar and two environmental conditions, the taxonomic abundance profiles were used to compute the Bray–Curtis distance matrix and plotted into two dimensions using nonmetric multidimensional scaling (NMDS). To understand the relative influence of each factor and their interaction on the overall phyllosphere microbial community structure, we performed a PERMANOVA on Bray–Curtis dissimilarities using cultivar, time of sampling, environment, and inoculation as independent variables. Overall, the effect of cultivar, time of sampling, and inoculation were highly significant in shaping bacterial communities (*p* < 0.001) in addition to the interactions of cultivar, time, and inoculation *(p* = 0.03) (Table S5A), with separation of inoculated susceptible plants from control susceptible, inoculated and control resistant plants (Fig. 3A). We further assessed individual factors’ influence and interactions across two sampling points. The effect of the cultivar was significant but diminished over the growing season (mid-season: R^2^ = 0.23, *p* < 0.001; end of the season: R^2^ = 0.06, *p* = 0.03). In contrast, effect of inoculation increased over the course of growing season (mid-season: R^2^ = 0.20, *p* < 0.001; end of the season: R^2^ = 0.55, *p* < 0.001) (Table S5B, C). The effect of interaction among cultivar and inoculation on bacterial communities remained statistically significant over time, although the effect decreased in size by the end of the growing season (mid-season: R^2^ = 0.15, *p* < 0.01; end of the season: R^2^ = 0.05, *p* = 0.04). The effect of elevated O_3_ was minimal, with it being not statistically significant by the end of the growing season (mid-season: R^2^ = 0.05 *p* = 0.04; end of the season: R^2^ = 0.02, *p* = 0.15) (Table S5B, C). The interaction between the environment and other variables was not statistically significant throughout the growing season. An increase in O_3_ levels did not alter the bacterial community structure on the susceptible cultivar. However, it influenced bacterial communities on the resistant cultivar (R^2^ = 0.14, *p* = 0.02) (Table S5D) in the absence of *Xanthomonas*. There was no difference in the microbial communities between the chambers with elevated O_3_ (*p* = 0.69) or ambient environment (*p* = 0.85) suggesting there is no effect of chamber in overall bacterial diversity (Table S5E, F).

Like bacterial communities, eukaryotic communities diversity was also significantly influenced by the environment, cultivar, time of sampling, and inoculation (*p* < 0.01) (Table S6A, B). Cultivar had a significant effect on eukaryotic diversity with more influence during the end of the season (mid-season: R^2^ = 0.12 (Table S6C), *p* = 0.007; end of the season: R^2^ = 0.37, *p* = 0.001 (Table S6D, E)). An increase in O_3_ levels significantly affected the eukaryotic communities during the mid-season, while it was not significant during the end of the season (mid-season: R^2^ = 0.22, *p* = 0.001 (Table S6C); end of the season: R^2^ = 0.06, *p* = 0.19 (Table S6D, E). The effect of inoculation on eukaryotic communities was higher during the mid-season, and it decreased during the end of the season (Fig. 3B) (mid-season: R^2^ = 0.15, *p* = 0.003 (Table S6C); end of the season: R^2^ = 0.11 *p* = 0.03 (Table S6D, E)). The influence of time of sampling on clustering was evident in shaping both bacterial and eukaryotic communities (Fig. 3A, B).

These findings indicate that microbial communities on resistant and susceptible cultivars were similar in absence of any stress, either pathogen or elevated O_3_, and influence of seasonal succession was evident on both bacterial and eukaryotic communities. Pathogen infection led to a shift in the bacterial community composition on the susceptible cultivar as the growing season progressed. However, despite presence of the *Xanthomonas* population on resistant cultivar, microbial community structure was like that observed on uninoculated plants. Despite increases in disease severity on the resistant cultivar under elevated O_3_, bacterial and eukaryotic communities were similar in their composition to that under ambient environment.

### Influence of pathogen infection and O_3_ stress on relative and absolute abundance of microbial taxa

The presence of *Xanthomonas* on control plants of susceptible and resistant cultivars suggested low levels of natural inoculum in the field. However, the relative abundance of *Xanthomonas* on control plants did not increase significantly over time (<5% by the end of the season). Both relative and absolute abundance of *Xanthomonas* increased from mid-season to end of the season on inoculated susceptible and resistant cultivars (Fig. S2A, B). Significant variation in the relative (~33–87%) as well as absolute (~13–37%) abundance of *Xanthomonas* on resistant inoculated plants under elevated O_3_ conditions was worth noting. However, presence of elevated O_3_ did not result in a significant difference in relative (Kruskal–Wallis: *p*_ECW_ = 0.12, *p*_X10R_ = 0.78) or absolute (Kruskal–Wallis: *p*_ECW_ = 0.15, *p*_X10R_ = 0.54) abundance of *Xanthomonas* in either cultivar (Fig. S2A, B). This observation was surprising given that disease severity levels under elevated O_3_ conditions on resistant inoculated plants were significantly higher than that under ambient environment.

To further confirm the influence of elevated O_3_ and cultivars on *Xanthomonas* population, we analyzed the *in planta* population of *X. perforans* determined using a culture-dependent method for day 7 and day 14 post-inoculation. While this short-course experiment may not reflect the outcome for the entire growing season, it allowed us to evaluate the effect of elevated O_3_ on the *Xanthomonas* population. Similar to the above observations, there was no significant effect of environment (i.e., ambient vs. elevated O_3_) on *X. perforans* population in these cultivars (*p*_ECW_ = 0.31, *p*_X10R_ = 0.34) (Fig. S2C).

**Fig. 4:**
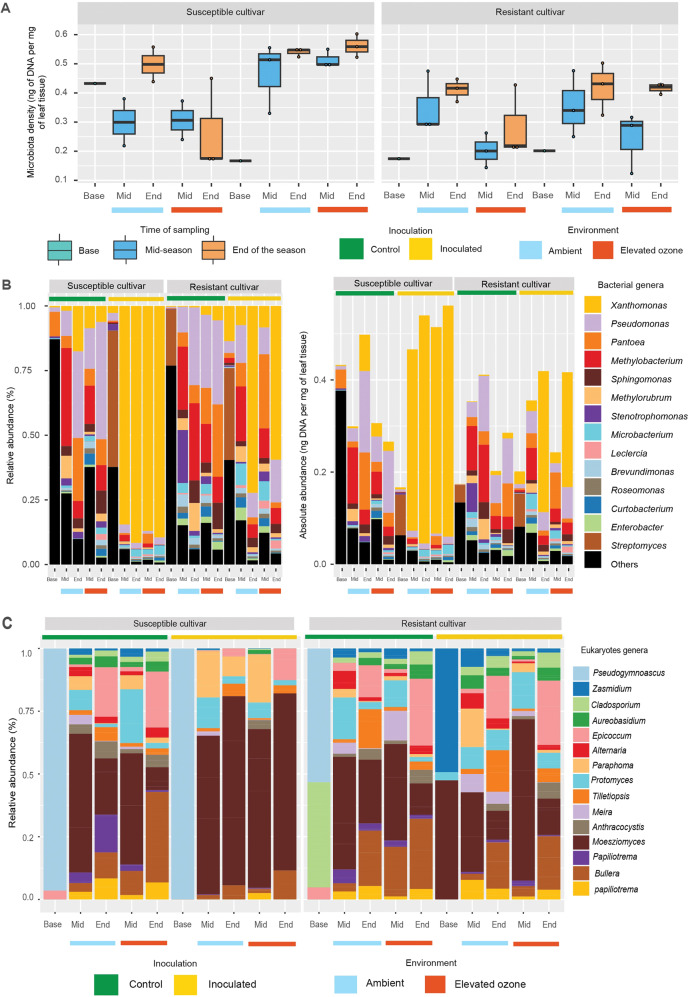
The effects of elevated O_3_ on disease outcomes are not fully explained by changes in microbiota density and abundance. **A** Box and whisker plot showing microbiota density estimated by microbial DNA quantification (concentration of extracted DNA per mg of leaf samples) for various treatment in two cultivars. **B** Relative (Left) and absolute (right) species abundance of top 15 bacterial taxa across samples. Absolute abundance is obtained by scaling the relative abundance measurements by the microbiota density measurements. **C** Bar plots showing the relative abundance of the top 15 eukaryotic genera across the samples. Inoculated and control samples are indicated with yellow and green bars on the top, while ambient and elevated O_3_ treatments are denoted by light blue and red color bars at the bottom, respectively. The time of sampling is indicated by Base (initial samples), Mid (mid-season), and End (end of the season).

As the increase in the disease severity on the resistant cultivar under elevated O_3_ was not the result of changes in *Xanthomonas* population, we hypothesized that this increase was associated with a significant reduction in overall microbial density associated with the resistant cultivar under elevated O_3_ compared to ambient environment, referring to an altered prophylactic shield from microbiota under elevated O_3_. Microbiota density estimates were obtained based on microbial DNA content per mg of sample, similar to those calculated in gut microbiome studies [77]. There was a significant effect of inoculation on microbiota density (*p* < 0.001), while neither cultivar (*p* = 0.15) nor elevated O_3_ (*p* = 0.19) had a significant effect on microbiota density (Fig. 4A). There was significantly lower microbiota density in mid-season samples on inoculated resistant cultivar under elevated O_3_ compared to susceptible cultivar (*p* = 0.01), but not for end of the season samples (*p* = 0.13) (Table S7A–D). We further estimated absolute abundance of each bacterial genus by multiplying its relative abundance (Fig. 4B) by the total DNA per mg of sample. Overall absolute abundance of microbiota was lower on inoculated resistant cultivar compared to inoculated susceptible cultivar, under both environments, although this difference was not statistically significant, accounting for large variation across samples (Fig. 4C, Table S7E, F). Total absolute abundance of microbiota associated with inoculated resistant cultivar under ambient environment was not significantly different compared to that under elevated O_3_ environment.

Next, we investigated the temporal dynamics in community assembly and succession in the phyllosphere, and patterns were compared between inoculated and control plants. Detailed taxonomic description of bacteria and eukaryotes across different treatments is given in supplementary information. The taxonomic diversity analysis showed that several bacterial (Fig. 4B, Table S8) and eukaryotic genera (Fig. 4C, Table S9) monopolizing the phyllosphere environment. These microbial genera are differentially affected by the presence of a pathogen, environmental stress, and their interaction.

Next, we identified genera that showed changes in relative abundance in response to cultivars, or elevated O_3_. Bacterial genera *Pseudomonas*, *Pantoea*, *Methylobacterium*, *Sphingomonas*, *Methylobrum*, etc., were negatively affected, while *Microbacterium* was positively influenced in the presence of *Xanthomonas* on susceptible cultivar (Fig. S3A–F). In contrast to the susceptible cultivar, the relative abundance of *Pseudomonas* and *Sphingomonas* increased in the presence of the *Xanthomonas* on the resistance cultivar. The bacterial genus *Methylobacterium* was negatively influenced by elevated O_3_, while the genera *Pseudomonas* and *Sphingomonas* were positively impacted in resistant cultivar (Fig. S3G–L). Regarding eukaryotes, the genus *Bullera* was positively affected by elevated O_3_, while the genus *Epicoccum* and *Protomyces* had temporal variation regardless of treatment (Fig. 4C).

### Functional composition of phyllosphere communities when exposed to O_3_ stress and pathogen infection

**Fig. 5:**
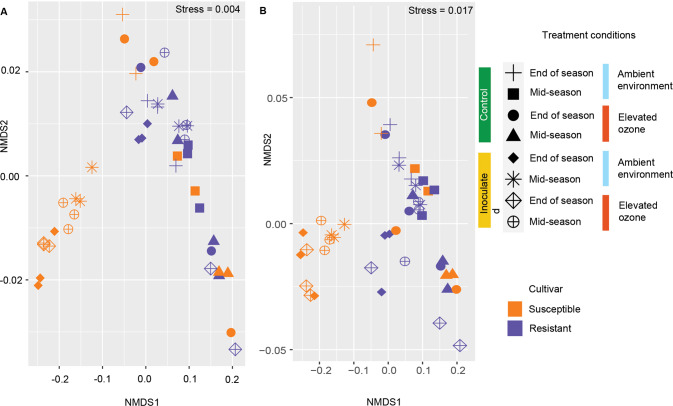
Microbial community functions were affected by host susceptibility to pathogens, while elevated O_3_ had little impact. Nonmetric Multidimensional Scaling (NMDS) ordination displaying diversity in **A** metabolic pathways across different treatment conditions in susceptible and resistant cultivars, **B** genes mapped to metabolic pathways across various treatment conditions in susceptible and resistant cultivars.

As the microbial composition was significantly affected by cultivar, inoculation, and time, we sought to investigate whether observed taxonomic differences reflected niche-specific microbial functions. Overall community functions based on the relative abundance of metabolic pathways (Fig. 5A) as well as associated gene families that were mapped onto the pathways (Fig. 5B) were not affected by these individual factors (*p* > 0.05) (Table S10A, B). However, the interaction between inoculation, cultivar, and sampling time had a significant effect on microbial functions and gene families (*p* < 0.01) (Table S10A, B), as indicated by dissimilarities in the functional composition of both gene families and pathways associated with communities recovered from the inoculated susceptible cultivar compared to the inoculated resistant cultivar. We observed significant effect of cultivar during the end of the season (*p* = 0.01) (Table S10C). Elevated O_3_ did not alter the functional assemblage of phyllosphere microbiome either on resistant or susceptible cultivars and regardless of the inoculation status. We observed similar functional profiles both in terms of genes as well as pathways across timepoints during the growing season on the respective cultivars despite differences in the species composition in mid vs. end of the season samples. This is likely due to substantial functional redundancy in the metabolic pathways associated with microbial communities over the growing season despite seasonal succession of taxa in the phyllosphere.

To find differentially abundant pathways that explain differences among treatments in response to pathogen infection, elevated O_3,_ and their interaction, we performed Linear discriminant analysis Effect Size (LEfSe). Upon pathogen infection and elevated O_3_, metabolic pathways related to heme scavenging (source of bioavailable iron) were enriched in microbial communities recovered from the resistant cultivar whereas pathways associated with carbohydrate metabolism (pentose phosphate pathway, gallate degradation, glyoxylate cycle), protection (lipid IV_A_ biosynthesis), growth and maintenance (phosphatidyl glycerol biosynthesis, CDP-diacylglycerol biosynthesis, GDP-mannose biosynthesis), and metabolism of unsaturated fatty acid (gondoate biosynthesis) were enriched in microbial communities recovered from the resistant cultivar under ambient conditions (Fig. S4A). Metabolic pathways that were enriched in microbial communities associated with both the cultivars upon O_3_ stress included pathways involved in primary energy production and the degradation of unsaturated fatty acids (beta-oxidation, pentose phosphate), various defense-related pathways against oxygen stress and DNA repair (ubiquinol 7, pyrimidine (deoxy)nucleotides) and pathways related to oxygen-independent respiration (oxygen-independent heme b biosynthesis) (Fig. S4B). In the presence of both the pathogen and elevated O_3_, pathway related to purine nucleotide production and degradation was enriched (Fig. S4C).

### Microbial network topology is altered under combined pathogen and O_3_ stress

**Fig. 6:**
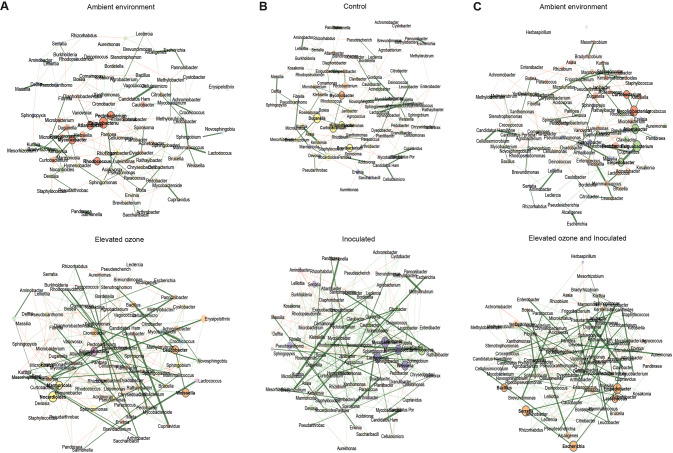
Pathogen infection is associated with microbial communities showing positive and stable interactions, but these interactions are random and less predictable with a shift in hub taxa in response to concurrent O_3_ stress and pathogen infection. Comparison of bacterial association network across different environments. **A** Bacterial association network for the combined data set of ambient (top) and elevated O_3_ (bottom) in both cultivars under control conditions. **B** Bacterial association network for the combined data set from control (top) and inoculated (bottom) samples from both the cultivars under ambient environment. **C** Bacterial association network for the combined data set from control and ambient environment (top) and inoculated and elevated O_3_ (bottom) samples from both cultivars. Hub taxa are highlighted by bold text. Node color represents the cluster determined by greedy modularity optimization. Red edges correspond to negative correlations, while green edges correspond to positive correlations.

To assess whether pathogen infection and O_3_ stress alone or in combination affected overall microbial association in the phyllosphere, bacterial co-occurrence networks and their topological features across treatments were compared. We assessed local network centrality measures using degree, betweenness, closeness and eigenvector centrality used to determine hub taxa for bacterial co-occurrence networks under elevated O_3_ (Fig. 6A), inoculation (Fig. 6B), and combined stress of elevated O_3_ and pathogen (Fig. 6C), and compared to ambient, control condition or control condition and ambient environment, respectively. We observed that all treatment comparisons mentioned above showed significant differences for all the local network centrality measures (Table S11A). A hub taxon is a highly connected taxon and is known to have strong impact in the network. There was a significant difference in hub taxa among treatment groups when comparing control with inoculated samples or control and ambient environment with pathogen and O_3_ stress (Table S11A, B). However, there was no change in hub taxa on plants exposed to elevated O_3_ compared to ambient environment.

Comparing the overall similarities of the two networks between the ambient vs. individual stress or combined stress of elevated O_3_ and pathogen based on adjusted Rand index (ARI) indicated values close to 0 (ARI = 0.02, *p* = 0.07) for ambient vs. elevated O_3_ stress; control vs. inoculated (ARI = 0.03, *p* = 0.02) and control and ambient environment vs combined stress (ARI = 0.10, *p* < 0.001) (Table S11C). These observations indicate that the partitions of species into communities show a low degree of similarity in these comparisons. These results, with differences in topology between these networks and dissimilarity in local network centrality measures, indicate that combination of pathogen infection and O_3_ stress results shifts in the bacterial community interactions in the phyllosphere.

Next, we assessed global microbial network properties such as number of edges as a measure of complexity, modularity, average path length and clustering coefficient, that compare network topologies across treatments [78, 79]. The current version of *NetCoMi* can only perform 1000 permutations due to the high run time of a single network construction. Since the minimum possible *p* value for 1000 permutations is 1/1000, the power is quite low, and this results in large *p* values after adjusting for multiple testing. Increasing number of permutations may allow evaluating global network properties with sufficient statistical power. Thus, in this study, we focused on absolute differences for each parameter under comparison, rather than associated *p* values. Microbial networks under ambient environment showed higher positive edge percentage, higher clustering coefficient, and lower average path length compared to elevated O_3_ (Table S11D). This suggests more positive interactions in ambient environments and that O_3_ stress may foster less complex and negative associations among community members. On the contrary, the presence of pathogen infection led to a more positive edge percentage, lower path length, higher modularity, and higher clustering coefficient, suggesting that all nodes were highly interlinked within the networks to form a more complex and stable network under pathogen infection (Table S11D). However, in the presence of pathogen infection and O_3_ stress, more positive interactions were found under ambient environment and control conditions with lower path length and higher clustering coefficient, suggesting that combined stress possibly creates less complex and less stable associations among community members (Table S11D).

## Discussion

Changing climate and modern agricultural practices have pre-disposed agro-ecosystems to an increased threat of pests, thus, leaving us with the unpredictability as to how plants will adapt to the simultaneous biotic and abiotic stressors. Many studies have proposed the role of plant-associated microbiomes in contributing towards plant resilience in the changing climate and extending plant immunity against pathogens [24, 30, 80, 81]. However, we have yet to fully understand how microbial communities, both respond to as well as contribute towards plant adaptation, in presence of simultaneous biotic and abiotic stressors. In this study, we tested individual and simultaneous effects of elevated O_3_ and pathogen stress on phyllosphere bacterial and eukaryotic community structure, function, and stability, and on overall plant disease outcomes on susceptible and resistant pepper cultivars. The resistant pepper cultivar used in this study possesses resistance genes that provide an intermediate level of resistance against all currently known pepper races of bacterial spot *Xanthomonas* [82]. Our rationale of including this cultivar in this study design was to understand the durability of this resistant cultivar that is currently widely deployed in the southeastern US in response to emerging pathogen species and under elevated O_3_, representing future climate.

While the apparent influence of elevated O_3_ was not observed on disease severity levels on the susceptible cultivar, the resistant cultivar displayed higher disease severity under elevated O_3_ throughout the growing season as compared to ambient environment (Fig. 1). This change in disease severity may also be indicative of resistance erosion under elevated O_3_ conditions. Unfortunately, the choice of cultivars used in this study not being near-isogenic prevents us from evaluating the influence of resistance loci on microbiome as was done in previous studies [83]. The increased disease severity observed on the resistant cultivar under elevated O_3_, however, was not associated with the increase in *Xanthomonas* population as estimated by absolute abundance data when compared to the ambient environment. Such a culture-independent DNA sequencing method may not accurately indicate living pathogen cell count and may warrant confirmation of these findings with a culture-dependent pathogen population estimate or with methods such as Quantitative PCR (qPCR) [84, 85], digital droplet PCR [86, 87]. Although not for the entire growing season, we monitored the dynamics of the *Xanthomonas* population during a short-term 2-week experiment and the results supported the previous findings that *Xanthomonas* population was unaffected despite higher disease severity under elevated O_3_ on the resistant cultivar. Interestingly, high variability in the *Xanthomonas* population counts on the resistant cultivar under elevated O_3_ was worth noting. This may indicate a plastic response of the pathogen during adaptation to the resistant cultivar under altered environment.

A large body of work has indicated that climatic fluctuations can have a profound effect on the outcome of plant-pathogen interactions [88–90], which may result from alteration of the host environment via modification of host defense pathways, increased pathogen infection efficiency under altered environments, or alteration in the microbiome-provided extended immunity. These three plausible explanations are outlined below that could synergistically drive plant-pathogen-microbiome interactions and help to explain the observation from this study of potential resistance erosion under elevated O_3_ conditions.

Studies on plant’s response to a combination of abiotic and biotic stress have shown a unique and more complex response than that of individual stresses [38, 91–93]. The effect of combined stress is governed by various factors such as time, degree of stress, plant genotype, and other climatic or environmental factors, thus, not necessarily additive in nature [94]. Plants respond to biotic and abiotic stresses via complex yet overlapping defense signaling pathways [95, 96], with induction of the abscisic acid (ABA) pathway observed upon abiotic stress, which antagonizes the salicylic acid (SA) pathway involved in pathogen defense [97, 98]. Simultaneous stresses of pathogen infection and elevated O_3_ may result in altered host immune response on the resistant cultivar. Oxidative damage of the plant cuticle caused by elevated [O_3_] can increase exposure to pathogens, thus, impacting disease severity [99]. Complementing this current study with host transcriptomics will explain if such host defense alteration may be what explains the increased susceptibility on resistant cultivar in presence of elevated O_3_. Secondarily, increased pathogen virulence via increased effector output [89] under altered environment may explain increased disease severity in absence of significant increase in pathogen population. The increased variation in pathogen population could be due to either host plastic response or plasticity in pathogen population.

Third and the most important explanation for the observations from this study is the alteration in microbiome-mediated protection on the resistant cultivar in response to elevated O_3_ and pathogen infection. Microbial communities recruited by the resistant cultivar in the phyllosphere could have a protective role against the pathogen as it has been demonstrated in previous studies [13, 100] and this protective role may have been altered under elevated O_3_, which may have led to increased disease severity. The bacterial and eukaryotic community composition, structure and function on the susceptible cultivar did not differ in the absence of pathogen infection or elevated O_3_. However, bacterial community structure on the resistant cultivar were influenced by presence of elevated O_3_, but in absence of the pathogen. Whether such differential influence on microbial community structure is due to specific resistance loci remains to be determined since the cultivars that we investigated were not near-isogenic lines for the resistance loci. On the susceptible cultivar, the presence of pathogen infection caused a sizeable shift in the bacterial community structure and function, even though concurrent O_3_ stress did not further alter the microbiome structure and function. Although no significant shift in microbiome structure and function was observed on the resistant cultivar upon infection, overall microbiota density associated with infected resistant cultivar was lower compared to infected susceptible cultivar. Furthermore, concurrent O_3_ stress resulted in lower total microbiota density during mid-season sampling on the infected resistant cultivar. Whether such reduction reflects impaired prophylactic potential of microbiome associated with resistant cultivar under the combined impact of elevated O_3_ and pathogen infection remains to be investigated. Further experiments to assess the microbiome-mediated protection against pathogen can be designed using synthetic communities associated with the resistant cultivar, similar to the previous studies [101]. These experiments may provide opportunities to dissect the influence of altered environment on absolute abundance of individual members of the community and their interactions, and associated functional traits. Interestingly, our data did not reveal any influence of simultaneous stressors on functions of microbial communities associated with the resistant cultivar. This was surprising given the previous studies indicate enrichment of specific metabolic pathways under abiotic or biotic stressors [102–104].

Microbial function in the ecosystem is determined not just because of the number and composition of taxa but also the various positive, negative, direct, or indirect associations among the community members [105]. In response to the pathogen challenge, we observed network parameters indicative of a densely connected network. These findings of enhanced positive and complex association among the microbial communities upon pathogen infection have been observed in both the phyllosphere and endosphere [106–108]. Such densely connected network indicates cooperative association such as facilitation, mutualism or commensalism, and cross-feeding [79, 109]. Such connected networks, referred to as small-world networks [110], are hypothesized to harbor resistance toward disturbances. In contrast, microbial co-occurrence networks across O_3_ stress and simultaneous pathogen and O_3_ stress showed a similar trend of a relatively unstable random network compared to the control environment. This finding agrees with the notion that varying degrees of environmental stress disturb the stability of microbial communities [79]. The observation from the similarity of the most central node suggests that microbial communities are considerably different across different treatments. The presence of a pathogen and simultaneous pathogen and O_3_ stress considerably affected hub taxa. However, simultaneous stressors, but not individual stresses, had considerable influence on the most influential taxa, suggesting that plants respond to simultaneous stresses by changing the most influential microbial member in the random network. It would be interesting to dissect further the influence of individual cultivar and, thus influence of host defense responses on microbial community networks, as we observed a strong cultivar effect on community composition. However, the present study is limited in sample size, which does not allow sufficient power to compare the network structure across individual cultivars. As we observed that elevated O_3_ impacted eukaryotic communities more strongly than bacterial communities and pathogen infection impacted bacterial communities, influence on cross-kingdom interactions cannot be ruled out in this case. Nevertheless, the present study has limitations in determining how specific and concurrent stressors affect cross-kingdom interactions due to the absence of appropriate methods to evaluate relative abundance of eukaryotic communities using shotgun metagenome data. It is possible that elevated O_3_ will have an impact on cross-kingdom interactions, as has been shown with other abiotic stressors [30].

Overall, our study demonstrated that microbial communities respond to a change by not only altering community composition but also interactions among members and overall community function. This work provides a base for our understanding of the complex response of microbial communities and their interactions with the host genotype in response to a changing climate. As plants have evolved in association with their phyllosphere microbiome members, the community members identified in this study have shown to be particularly susceptible to a shift in response to abiotic stress or combined stress. Findings from this study are crucial to evaluate for future work on harnessing the microbiome for stress-tolerant plants.

## Supplementary Information

Supplementary materials

## Data Availability

Sequence data generated from this work have been deposited in the SRA (Sequencing Read Achieve) database under the BioProject accessions PRJNA889178. All other data and code used in this study are available in the following GitHub repository (https://github.com/Potnislab/AtDep_2021_metagenome).
