# Supplementary material for: Xanthomonas infection and ozone stress distinctly influence the microbial community structure and interactions in the pepper phyllosphere
Source: ISME Commun. 2023 Mar 27;3:24. doi: 10.1038/s43705-023-00232-w (PMC10043289; doi:10.1038/s43705-023-00232-w)
Supplement: Supplementary file 1 — Supplementary materials [file 43705_2023_232_MOESM1_ESM.pdf]

Supplementary results

Taxonomic profiling

A temporal pattern was observed in that, *Actinobacteria* were the most abundant (~57%) in the base samples, and their abundance decreased over time both in inoculated (mid-season: 8 %; end of the season: 1%) and control plants (mid-season: 9 %; end of the season: 3%) shifting to Proteobacteria which was dominant both at the mid and end of the season samples (Figure S5). Phylum Bacteroidetes, although accounting for around 3% in the base samples, decreased in abundance to less than 1% at later time points and were negligible on the inoculated susceptible leaves. Phylum Firmicutes contributed to around 2% of the sequences during the mid-season. They were in higher abundance under elevated O_3 conditions on the leaves of the control susceptible cultivar and in both inoculated and control plants in the resistant cultivar. This phylum was affected by inoculation on susceptible plants and was negligible across all the treatment conditions during the end of the season.

We next examined the deeper taxonomic ranks, specifically, genus, for further comparison of patterns affected by the presence of the pathogen and elevated O_3 or a combination of both stresses. Susceptible control plants were dominated mainly by *Pseudomonas* (~5-60%), *Pantoea* (~3-28%), and *Sphingomonas* (3-15%), while *Xanthomonas* (~84-98%) and *Pseudomonas* (~1-4%) were the dominant genera in the inoculated plants. In resistant cultivar, *Pseudomonas* (6-40%), *Pantoea* (~2-44%), and *Methylobacterium* (~9-35%) were the dominant genera in control plants, while *Xanthomonas* (~2-87%), *Pseudomonas* (~2-27%), and *Methylobacterium* (~1-26%) were dominant on inoculated plants (Fig. 4B, Table S8). Other genera contributing to the phyllosphere microbiome included *Pseudomonas*, *Pantoea*, *Methylobacterium, Sphingomonas, Methylorubrum*, *Stenotrophomonas,* and *Microbacterium*. The abundance of *Pseudomonas*

increased over the growing season, except for resistant control plants, where abundance levels were higher during the mid-season under elevated O_3 and stayed at similar levels throughout the growing season. *Pseudomonas* abundance was affected by inoculation, where a decrease in the abundance of *Pseudomonas* was proportional to the increase in *Xanthomonas* abundance on either cultivar. The mean relative abundance of *Pantoea* increased over time in control plants of either cultivar. The inoculation shifted the temporal pattern with a decreased mean relative abundance of *Pantoea* from mid to end of the season sampling. This decrease was statistically significant under elevated O_3 on inoculated samples of either cultivar (resistant cultivar: p = 0.03; susceptible cultivar: $p < 0.01$). Inoculation also had a slight effect on the mean relative abundance of *Methylobacterium* with a significant decrease with inoculation under ambient environment ($p < 0.01$), under elevated O_3 in the resistant cultivar (Kruskal-Wallis, $p = 0.04$), and ambient environment ($p = 0.02$) in the susceptible cultivar. An increase in O_3 concentration has a negative effect on the *Methylobacterium* genus under control conditions and ambient environment (resistant cultivar: $p < 0.01$, susceptible cultivar: p= 0.02). However, the environment did not affect the *Methylobacterium* genus in inoculated samples in both cultivars (Fig. S3).

Analysis of the eukaryotes diversity across our samples showed that the genera *Moesziomyces*, *Golubevia*, *Paraphoma, Protomyces,* and *Cercospora* were dominant across the samples (Fig. 4C, Table S9). Base samples were dominated by the genus *Pseudogymnoascus* (~64%), which was not observed during later time points. Genus *Bullera* had a higher relative abundance under elevated O_3 both in control (~ 32%) and inoculated plants (~16%) than that of ambient environment (~ 15% in control and 12% in inoculated) during the end of the season in both the cultivars. Inoculation resulted in lower *Zasmidium, Cladosporium, Aureobasidium, Alternaria,*

and *Anthracocystis* genera in susceptible cultivars compared to resistant ones under both environmental conditions. Regardless of treatments, the relative abundance of the genus *Epicoccum* increased at the end of the season (~ 31%) compared to mid-season (~ 4%). In comparison, the genus *Protomyces* decreased during the end of the season (~ 3%) compared to mid-season (~ 11%). Inoculation did not affect the relative abundance of *Moesziomyces* in susceptible cultivars during both mid (~ 60%) and end of the season (~70%). In comparison, the presence of *Xanthomonas* had a negative effect on this genus during the end of the season (~ 12%) in resistant cultivars.

Supplementary figure legends

Fig. S1. Study site and treatment design of Atmospheric Deposition Laboratory (AtDep) site at Auburn University. (A) Satellite image of AtDep site with individual chambers label where light blue circles are the chambers with the ambient environment and red circles are the chambers with elevated O_3. Chambers 1-6 marked with yellow color are inoculated with *X. perforans*, and chambers 7-12, with green color are control samples. (B) Individual open-top chamber at the AtDep site (4 x 5m). (C) Daily average O_3 concentration in treatment chambers. Sampling points (mid and end of the season) are marked by a red arrow. (D) Inoculation method showing how plants were inoculated with *Xanthomonas* and control plants with $MgSO_4$ buffer. (E) The plants were then kept inside the OTCs where each chamber had 12 plants (6 each from susceptible and resistant cultivar). Inoculated and control plants were kept in different chambers as per the treatment plan in A.

Fig. S2. Elevated O_3 increases heterogeneity in *Xanthomonas* population counts on the resistant cultivar. Line graph showing the relative and absolute abundance (ng of DNA per mg of leaf tissue) (A) *Xanthomonas* relative and absolute abundance (ng of DNA per mg of leaf tissue) across different treatments in susceptible cultivars. (B) *Xanthomonas* relative and absolute abundance (ng of DNA per mg of leaf tissue) across different treatments in resistant cultivar. end of the season. (C) *Xanthomonas* population growth based on culture-dependent technique on two different cultivars under ambient environment and elevated O_3.

Fig. S3. Box plot showing the relative abundance of top 6 bacterial genera. (A-F) Relative abundance of different bacterial genera on susceptible cultivar across different treatments; (G-L) Relative abundance of different bacterial genera on resistant cultivar across different treatments. Control samples are indicated by a green bar, while the yellow bar represents the inoculated

samples. The blue box represents the samples taken during the mid-season, while the orange box is end of the season samples. Different environmental conditions are represented by the ambient environment and elevated O_3 under both inoculation and control treatments.

Fig. S4. Linear Discriminant Analysis Effect Size (LEfSe) of KEGG pathways between susceptible and resistant cultivar. Results were ranked by their Linear Discriminant Analysis (LDA) score. An FDR-adjusted *p*-value ≤ 0.05, as well as an LDA score ≥2.5, were used as thresholds to identify significant features. (A) pathways enriched upon inoculation of the resistant cultivar under elevated O_3 (brown), resistant cultivar under ambient environment (violet), susceptible cultivar under elevated O_3 (cyan), and susceptible cultivar under ambient environment (black). (B) pathways enriched in both cultivars under elevated O_3 (red) and ambient environment (blue), (C) pathways enriched in both the cultivars under combined stress of pathogen infection and elevated O_3 (orange) and ambient environment (lime green).

Fig. S5. Stacked bar plots showing the relative abundance of dominant phyla across the samples. Control samples are indicated by a green bar, while the yellow bar represents the inoculated samples. The time of sampling is indicated by Base (initial samples), Mid (mid-season) and End (end of the season).

Supplementary table legends

Table S1: Multiple pairwise comparisons (Dunn test) of disease severity index across different cultivars and treatment conditions. Significance levels for each treatment combination are indicated by *$p < 0.05$; **$p < 0.01$; ***$p < 0.001$; ****$p < 0.0001$.

Table S2: Sample details and sequencing statistics of metagenome reads.

Table S3A: Pairwise comparison for bacterial Chao1 diversity index for resistant pepper. Significance levels for each treatment combination are indicated by *$p < 0.05$; **$p < 0.01$; ***$p < 0.001$.

Table S3B: Pairwise comparison for bacterial Shanon diversity index(Dunn's multiple comparison test) for resistant pepper. Significance levels for each treatment combination are indicated by *$p < 0.05$; **$p < 0.01$; ***$p < 0.001$.

Table S3C: Pairwise comparison for bacterial Chao1 diversity index for susceptible pepper. Significance levels for each treatment combination are indicated by *$p < 0.05$; **$p < 0.01$; ***$p < 0.001$.

Table S3D: Pairwise comparison for bacterial Shanon diversity index (Kruskal-Wallis multiple comparisons & p-values adjusted with the Benjamini-Hochberg method) for control susceptible pepper.

Table S3E: Pairwise comparison for bacterial Shanon diversity index for inoculated susceptible pepper. Significance levels of treatment combination are indicated by *$p < 0.05$; **$p < 0.01$; ***$p < 0.001$.

Table S4A: Pairwise comparison of Eukaryotic Chao1 diversity index for resistant pepper. Significance levels for each treatment combination are indicated by *$p < 0.05$; **$p < 0.01$; ***$p < 0.001$.

Table S4B: Pairwise comparison of Eukaryotic Shannon diversity index for resistant pepper. Significance levels for each treatment combination are indicated by *$p < 0.05$; **$p < 0.01$; ***$p < 0.001$.

Table S4C: Pairwise comparison of Eukaryotic Chao1 diversity index for susceptible pepper. Significance levels for each treatment combination are indicated by *$p < 0.05$; **$p < 0.01$; ***$p < 0.001$; ****$p < 0.0001$.

Table S4D: Pairwise comparison of Eukaryotic Shanon diversity index for susceptible pepper. Significance levels for each treatment combination are indicated by *$p < 0.05$; **$p < 0.01$; ***$p < 0.001$.

Table S5A: Permutational multivariant analysis of variance (PERMANOVA) of bacterial community composition based on Bray–Curtis dissimilarities of their relative abundance. Significance levels for each treatment combination are indicated by *$p < 0.05$; **$p < 0.01$; ***$p < 0.001$.

Table S5B: Permutational multivariant analysis of variance (PERMANOVA) of bacterial community composition based on Bray–Curtis dissimilarities of relative abundance during the mid-season. Significance levels for each treatment combination are indicated by *$p < 0.05$; **$p < 0.01$; ***$p < 0.001$.

Table S5C: Permutational multivariant analysis of variance (PERMANOVA) of bacterial community composition based on Bray–Curtis dissimilarities of relative abundance during end of the season. Significance levels for each treatment combination are indicated by $^{*}p < 0.05$; $^{**}p < 0.01$; $^{***}p < 0.001$.

Table S5D: Permutational multivariant analysis of variance (PERMANOVA) of bacterial community composition based on Bray–Curtis dissimilarities of relative abundance in control samples of the resistant cultivar. Significance levels for each treatment combination are indicated by $^{*}p < 0.05$; $^{**}p < 0.01$; $^{***}p < 0.001$.

Table S5E: Permutational multivariant analysis of variance (PERMANOVA) of bacterial community composition based on Bray–Curtis dissimilarities of relative abundance in samples from the elevated ozone. Significance levels for each treatment combination are indicated by $^{*}p < 0.05$; $^{**}p < 0.01$; $^{***}p < 0.001$.

Table S5F: Permutational multivariant analysis of variance (PERMANOVA) of bacterial community composition based on Bray–Curtis dissimilarities of relative abundance in samples from the ambient environment. Significance levels for each treatment combination are indicated by $^{*}p < 0.05$; $^{**}p < 0.01$; $^{***}p < 0.001$.

Table S6A: Permutational multivariant analysis of variance (PERMANOVA) of eukaryotes community composition based on Bray–Curtis dissimilarities of relative abundance. Significance levels for each treatment combination are indicated by $^{*}p < 0.05$; $^{**}p < 0.01$; $^{***}p < 0.001$.

Table S6B: Analysis of similarity (ANOSIM) of eukaryotes community composition based on Bray–Curtis dissimilarities of relative abundance influenced by different factors. Significance levels for each treatment combination are indicated by $*p < 0.05$; $**p < 0.01$; $***p < 0.001$.

Table S6C: Permutational multivariant analysis of variance (PERMANOVA) of eukaryote community composition based on Bray–Curtis dissimilarities of relative abundance during mid-season. Significance levels for each treatment combination are indicated by $*p < 0.05$; $**p < 0.01$; $***p < 0.001$.

Table S6D: Permutational multivariant analysis of variance (PERMANOVA) of eukaryote community composition based on Bray–Curtis dissimilarities of relative abundance during the end season. Significance levels for each treatment combination are indicated by $*p < 0.05$; $**p < 0.01$; $***p < 0.001$.

Table S6E: Analysis of similarity (ANOSIM) of eukaryotes community composition based on Bray–Curtis dissimilarities of relative abundance during end season. Significance levels for each treatment combination are indicated by $*p < 0.05$; $**p < 0.01$; $***p < 0.001$.

Table S7A: Permutational multivariant analysis of variance (PERMANOVA) microbiota density across samples during mid-season and ambient environment based on Bray–Curtis dissimilarities in Control samples. Significance levels for each treatment combination are indicated by $*p < 0.05$; $**p < 0.01$; $***p < 0.001$.

Table S7B: Pairwise comparison of microbiota density across samples during mid-season under elevated ozone. Significance levels for each treatment combination are indicated by $*p < 0.05$; $**p < 0.01$; $***p < 0.001$.

Table S7C: Permutational multivariant analysis of variance (PERMANOVA) microbiota density across samples during mid-season and ambient environment based on Bray–Curtis dissimilarities in Control samples. Significance levels for each treatment combination are indicated by $*p < 0.05$; $**p < 0.01$; $***p < 0.001$.

Table S7D: Pairwise comparison of microbiota density across samples during the end of the season and elevated ozone. Significance levels for each treatment combination are indicated by $*p < 0.05$; $**p < 0.01$; $***p < 0.001$.

Table S7E: Permutational multivariant analysis of variance (PERMANOVA) on the total absolute abundance of microbial communities based on Bray–Curtis dissimilarities in Control samples. Significance levels for each treatment combination are indicated by $*p < 0.05$; $**p < 0.01$; $***p < 0.001$.

Table S7F: Pairwise comparison of the total absolute abundance of microbial communities in inoculated samples (only the significant treatment combinations are shown in the table). Significance levels for each treatment combination are indicated by $*p < 0.05$; $**p < 0.01$; $***p < 0.001$.

Table S8: Relative abundance of top 18 bacterial genera across the samples based on Kraken2 and Bracken

Table S9: Normalized RPKS (Reads Per Kilobase of Sequence) value of eukaryotes genera across samples obtained from the EukDetect pipeline.

Table S10A: Permutational multivariant analysis of variance (PERMANOVA) of microbial pathways based on Bray–Curtis dissimilarities. Significance levels for each treatment combination are indicated by *$p < 0.05$; **$p < 0.01$; ***$p < 0.001$.

Table S10B: Permutational multivariant analysis of variance (PERMANOVA) of microbial genes based on Bray–Curtis dissimilarities. Significance levels for each treatment combination are indicated by *$p < 0.05$; **$p < 0.01$; ***$p < 0.001$.

Table S10C: Permutational multivariant analysis of variance (PERMANOVA) of microbial pathways based on Bray–Curtis dissimilarities during mid and end of the season. Significance levels for each treatment combination are indicated by *$p < 0.05$; **$p < 0.01$; ***$p < 0.001$.

Table S11A: Comparison of jaccard index (similarity between the sets of most central nodes) of local network centrality measures between groups. Significance levels for each treatment combination are indicated by *$p < 0.05$; **$p < 0.01$; ***$p < 0.001$.

Table S11B: Microbial hub taxa across treatment groups

Table S11C: Adjusted Rand index (similarity between clustering) across treatment groups

Table S11D: Topological features of the taxonomic networks across various treatments

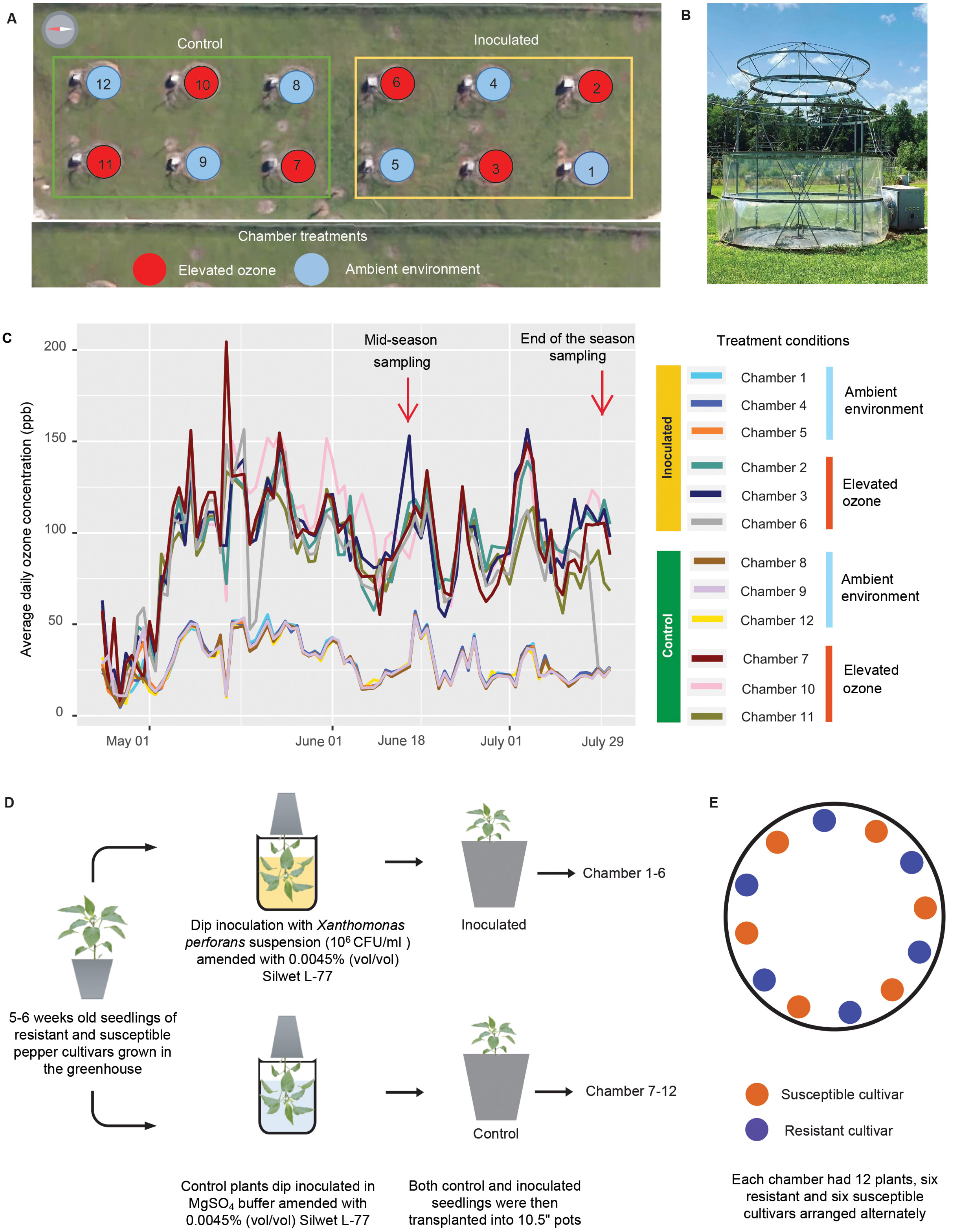

Fig. S1. Study site and treatment design of Atmospheric Deposition Laboratory (AtDep) site at Auburn University. (A) Satellite image of AtDep site with individual chambers label where light blue circles are the chambers with the ambient environment and red circles are the chambers with elevated O_3. Chambers 1-6 marked with yellow color are inoculated with X. perforans, and chambers 7-12, with green color are control samples. (B) Individual open-top chamber at the AtDep site (4 x 5m). (C) Daily average O_3 concentration in treatment chambers. Sampling points (mid and end of the season) are marked by a red arrow. (D) Inoculation method showing how plants were inoculated with *Xanthomonas* and control plants with $MgSO_4$ buffer. (E) The plants were then kept inside the OTCs where each chamber had 12 plants (6 each from susceptible and resistant cultivar). Inoculated and control plants were kept in different chambers as per the treatment plan in A.

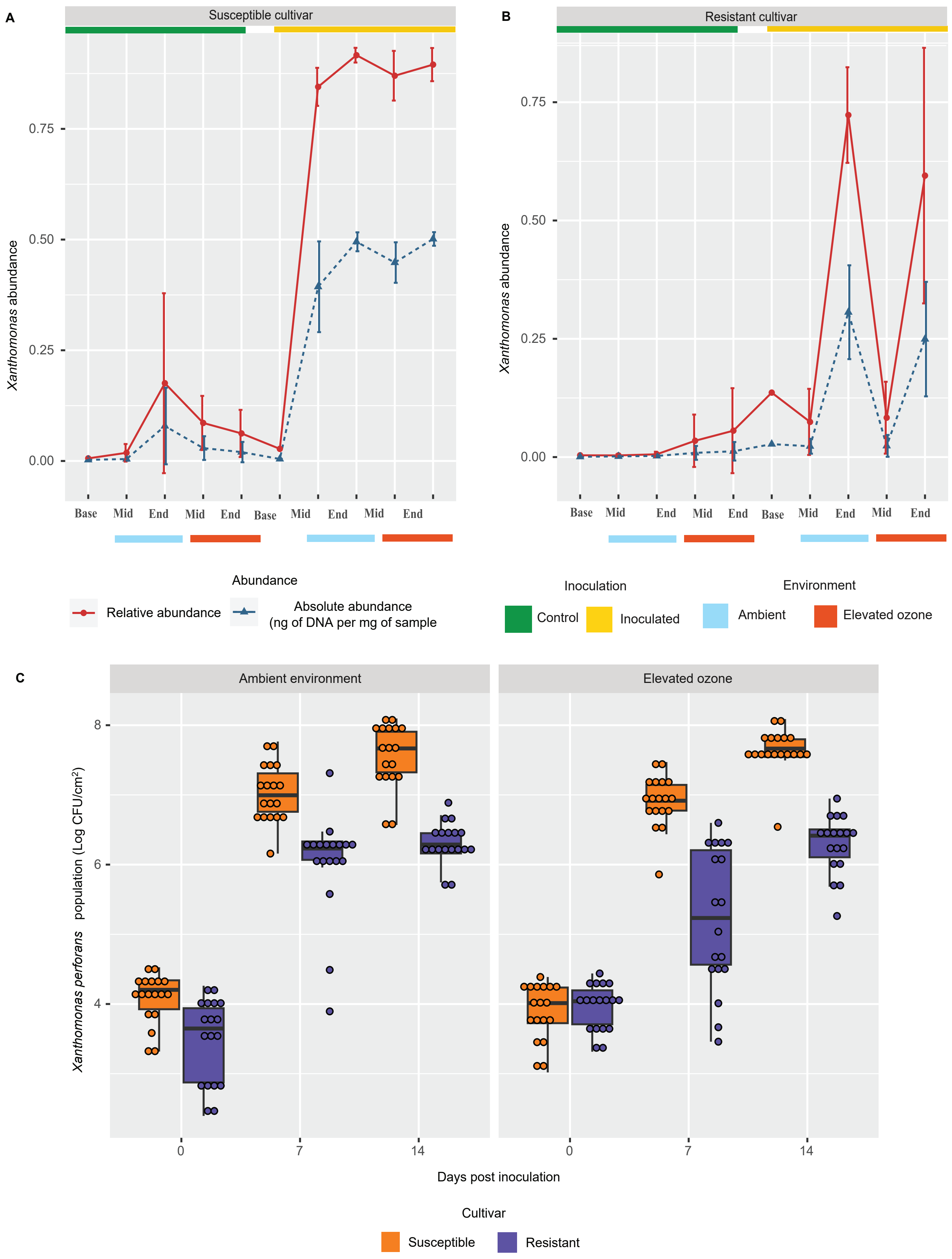

Fig. S2. Elevated O_3 increases heterogeneity in *Xanthomonas* population counts on the resistant cultivar. Line graph showing the relative and absolute abundance (ng of DNA per mg of leaf tissue) (A) *Xanthomonas* relative and absolute abundance (ng of DNA per mg of leaf tissue) across different treatments in susceptible cultivars. (B) *Xanthomonas* relative and absolute abundance (ng of DNA per mg of leaf tissue) across different treatments in resistant cultivar. (C) *Xanthomonas* population growth based on culture-dependent technique on two different cultivars under ambient environment and elevated O_3.

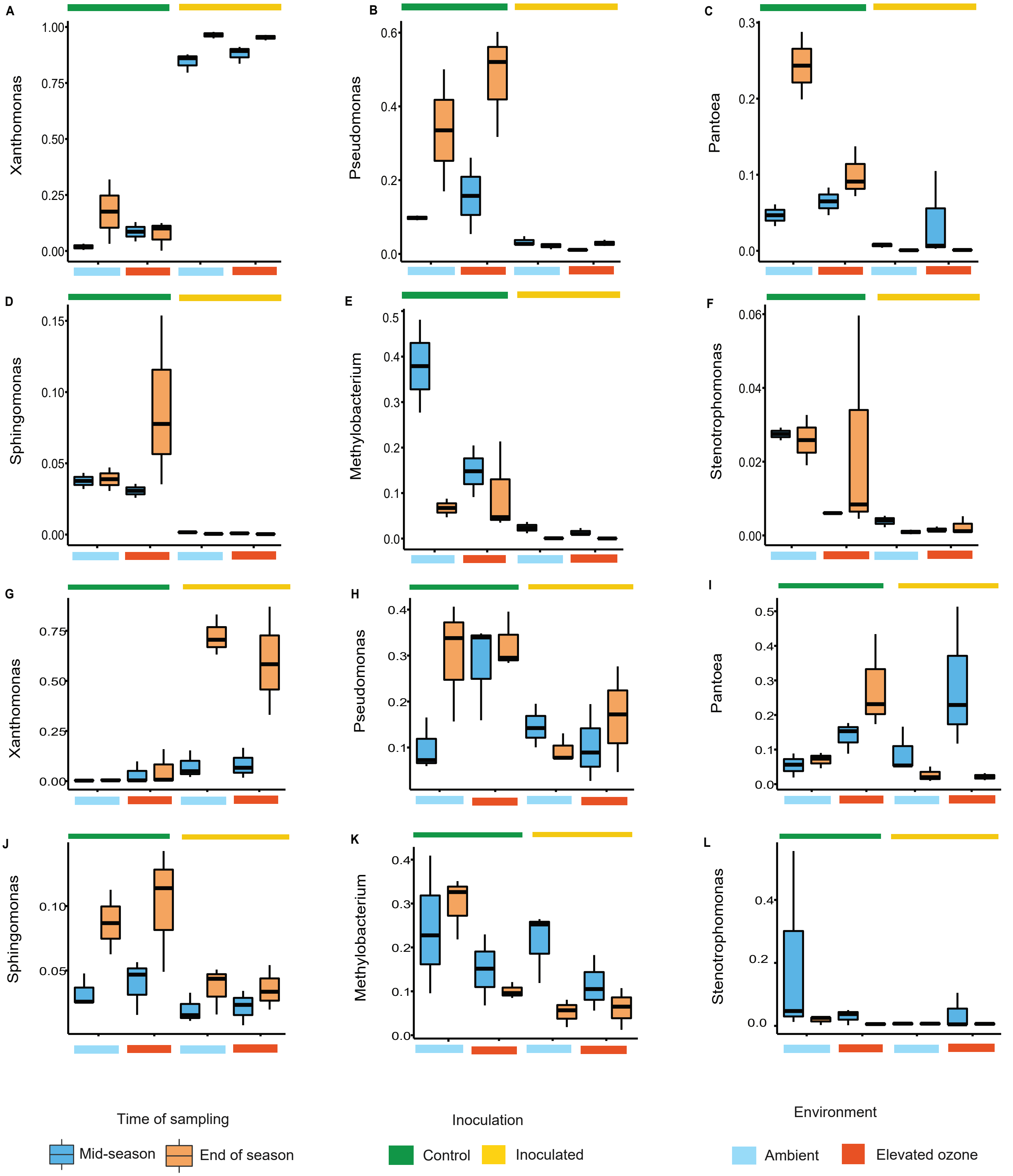

Fig. S3. Box plot showing the relative abundance of top 6 bacterial genera. (A-F) Relative abundance of different bacterial genera on susceptible cultivar across different treatments; (G-L) Relative abundance of different bacterial genera on resistant cultivar across different treatments. Control samples are indicated by a green bar, while the yellow bar represents the inoculated samples. The blue box represents the samples taken during the mid-season, while the orange box is end of the season samples. Different environmental conditions are represented by the ambient environment and elevated O_3 under both inoculation and control treatments.

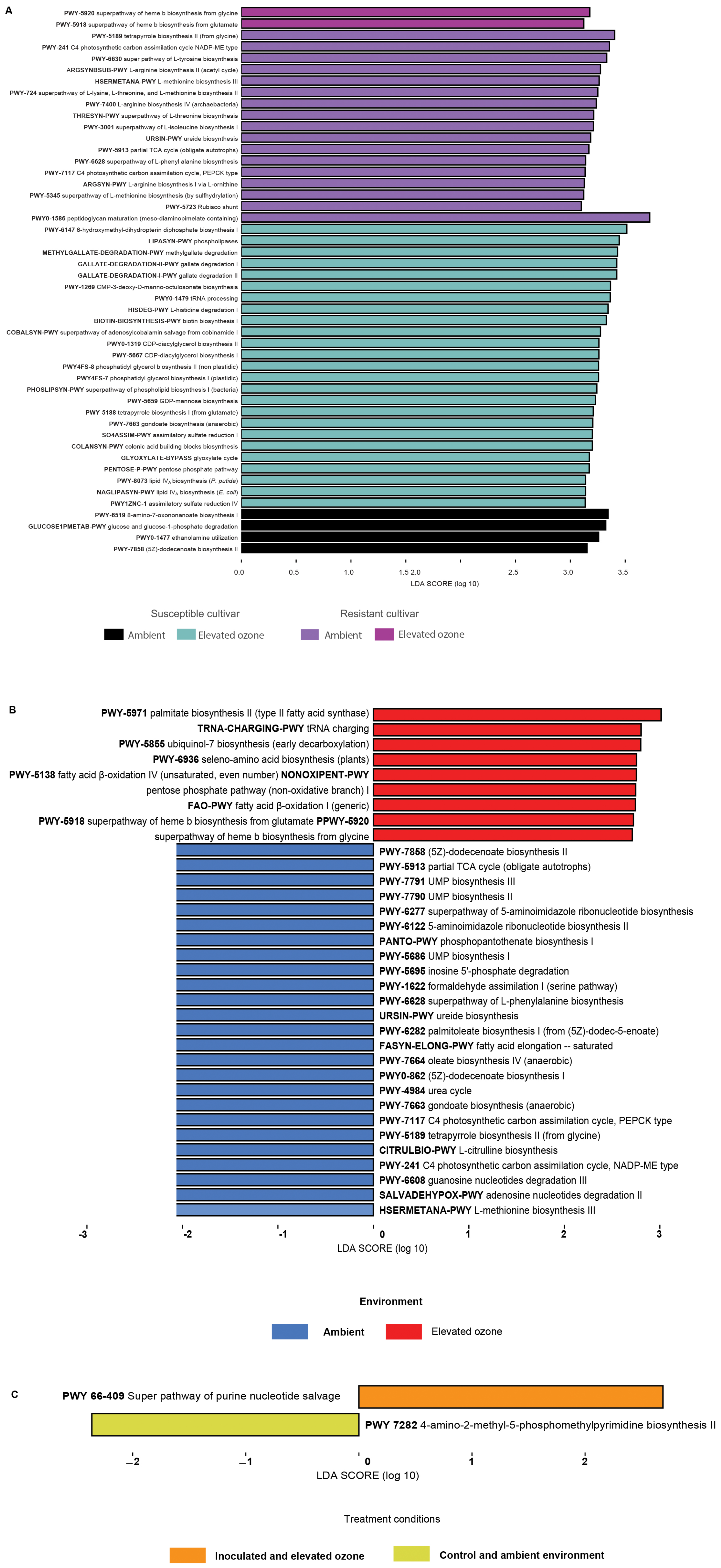

Fig. S4. Linear Discriminant Analysis Effect Size (LEfSe) of KEGG pathways between susceptible and resistant cultivar. Results were ranked by their Linear Discriminant Analysis (LDA) score. An FDR-adjusted *p*-value ≤ 0.05, as well as an LDA score ≥ 2.5, were used as thresholds to identify significant features. (A) Pathways enriched upon inoculation of the resistant cultivar under elevated O_3 (brown), resistant cultivar under ambient environment (violet), susceptible cultivar under elevated O_3 (cyan), and susceptible cultivar under ambient environment (black). (B) Pathways enriched in both cultivars under elevated O_3 (red) and ambient environment (blue), (C) pathways enriched in both the cultivars under combined stress of pathogen infection and elevated O_3 (orange) and ambient environment (lime green).

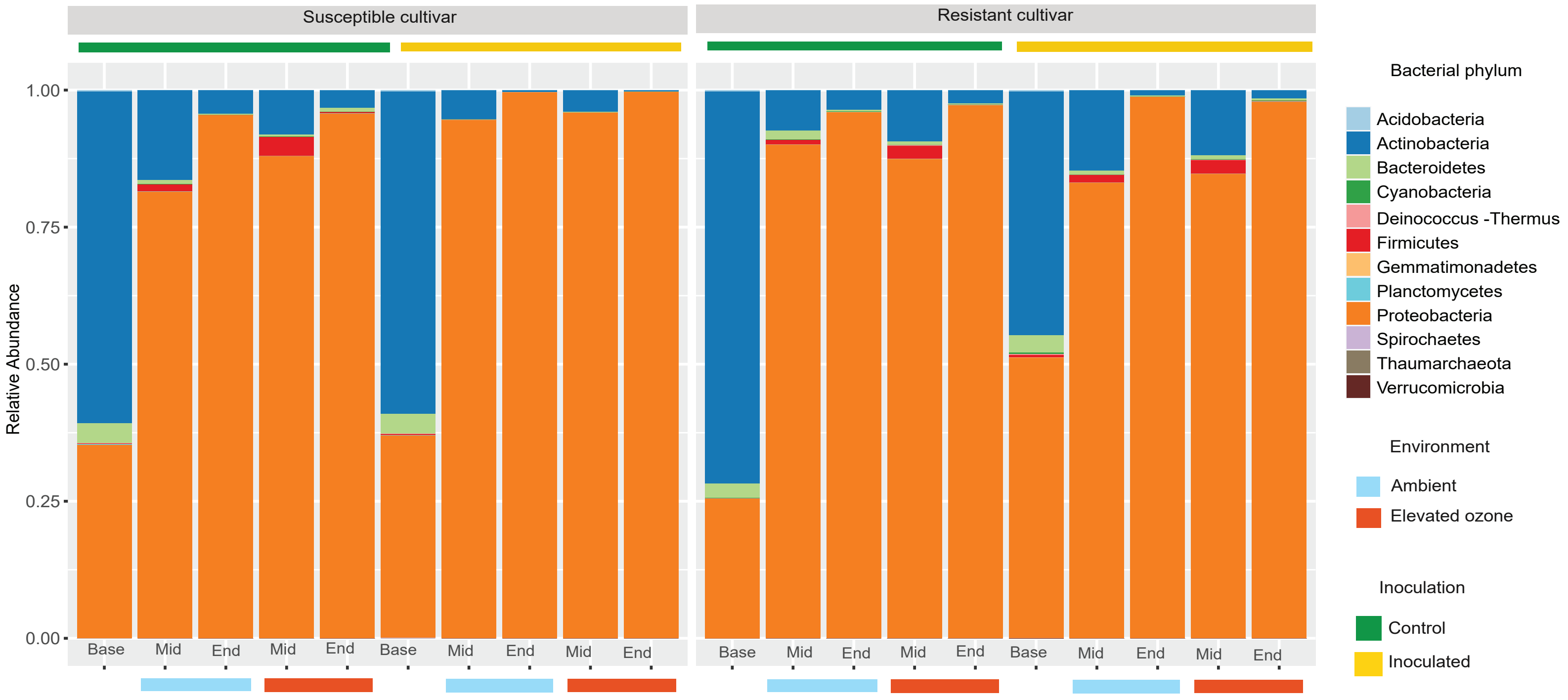

Fig. S5. Stacked bar plots showing the relative abundance of dominant phyla across the samples. Control samples are indicated by a green bar, while the yellow bar represents the inoculated samples. The time of sampling is indicated by Base (initial samples), Mid (mid-season) and End (end of the season).

Table S1: Multiple pairwise comparisons (Dunn test) of disease severity index across different cultivars and treatment conditions. Significance levels for each treatment combination are indicated by *p < 0.05; **p < 0.01; ***p < 0.001; ****p < 0.0001.

Cultivar	Comparison	Standard test statistics	*p* value	Adjusted *p*-value	significance
Susceptible	Ambient environment and end of the season vs. Ambient environment and mid-season	4.9074802	9.23E-07	5.54E-06	****
Susceptible	Ambient environment and end of the season vs. Elevated ozone and end of the season	0.8719077	3.83E-01	3.83E-01	
Susceptible	Ambient environment and end of the season vs. Elevated ozone and mid-season	3.4676327	5.25E-04	1.05E-03	**
Susceptible	Ambient environment and mid-season vs. Elevated ozone and end of the season	-4.0355726	5.45E-05	1.63E-04	***
Susceptible	Ambient environment and mid-season vs. Elevated ozone and mid-season	-1.4398475	1.50E-01	1.80E-01	
Susceptible	Elevated ozone and end of the season vs. Elevated ozone and mid-season	2.5957251	9.44E-03	1.42E-02	*
Resistant	Ambient environment and end of the season vs. Ambient environment and mid-season	0.19131	8.48E-01	8.48E-01	
Resistant	Ambient environment and end of the season vs. Elevated ozone and end of the season	2.7448823	6.05E-03	9.08E-03	**
Resistant	Ambient environment and end of the season vs. Elevated ozone and mid-season	6.0969658	1.08E-09	6.49E-09	****
Resistant	Ambient environment and mid-season vs. Elevated ozone and end of the season	2.5535723	1.07E-02	1.28E-02	*
Resistant	Ambient environment and mid-season vs. Elevated ozone and mid-season	5.9056558	3.51E-09	1.05E-08	****
Resistant	Elevated ozone and end of the season vs. Elevated ozone and mid-season	3.3520835	8.02E-04	1.60E-03	**

Table S2: Sample details and sequencing statistics of metagenome reads.

Sample id	Cultivar	Inoculation status	Time of sampling	Environment	DNA conc. (ng/µl)	Total raw reads	Total raw bases	Reads after quality trimming	Reads after host trimming	% of final reads for further analysis	Gbp of reads	% lost in quality trimming	% lost in host trimming
1EM	Susceptible	Inoculated	Mid-season	Ambient	44.4	94632028	14012568232	82555398	76943654	81.31	14.01	12.76	6.80
1EE	Susceptible	Inoculated	End of the season	Ambient	43.8	71879368	10853784568	63532100	59509512	82.79	10.85	11.61	6.33
1XM	Resistant	Inoculated	Mid-season	Ambient	27.2	86444924	13053183524	75569647	70279794	81.30	13.05	12.58	7.00
1XE	Resistant	Inoculated	End of the season	Ambient	34.5	83058106	12541774006	73320424	68466214	82.43	12.54	11.72	6.62
2EM	Susceptible	Inoculated	Mid-season	Elevated ozone	44	83619784	12626587384	73105423	68279082	81.65	12.63	12.57	6.60
2EE	Susceptible	Inoculated	End of the season	Elevated ozone	48.2	76613288	11568606488	68214364	64256044	83.87	11.57	10.96	5.80
2XM	Resistant	Inoculated	Mid-season	Elevated ozone	23.1	67954684	10261157284	58945911	54317988	79.93	10.26	13.26	7.85
2XE	Resistant	Inoculated	End of the season	Elevated ozone	31.6	85486336	12908436736	74113643	61375640	71.80	12.91	13.30	17.19
3EM	Susceptible	Inoculated	Mid-season	Elevated ozone	39.5	78103064	11793562664	68629726	64244500	82.26	11.79	12.13	6.39
3EE	Susceptible	Inoculated	End of the season	Elevated ozone	44.7	82463524	12451992124	72822036	68362944	82.90	12.45	11.69	6.12
3XM	Resistant	Inoculated	Mid-season	Elevated ozone	9.88	72073444	10883090044	63021647	58620704	81.33	10.88	12.56	6.98
3XE	Resistant	Inoculated	End of the season	Elevated ozone	34.8	107486130	16230405630	96211494	84545768	78.66	16.23	10.49	12.13
4EM	Susceptible	Inoculated	Mid-season	Ambient	26.4	92419428	13955333628	81432726	76535500	82.81	13.96	11.89	6.01
4EE	Susceptible	Inoculated	End of the season	Ambient	43.9	60405554	9121238654	53906544	50787970	84.08	9.12	10.76	5.79
4XM	Resistant	Inoculated	Mid-season	Ambient	38.1	85511946	12912303846	74096844	68386118	79.97	12.91	13.35	7.71
4XE	Resistant	Inoculated	End of the season	Ambient	40.2	95167264	14370256864	82585708	72069602	75.73	14.37	13.22	12.73
5EM	Susceptible	Inoculated	Mid-season	Ambient	41.1	86126728	13005135928	75442752	70497018	81.85	13.01	12.40	6.56
5EE	Susceptible	Inoculated	End of the season	Ambient	41.9	69052192	10426880992	59171173	54881300	79.48	10.43	14.31	7.25
5XM	Resistant	Inoculated	Mid-season	Ambient	20	87321158	13185494858	76345143	71081610	81.40	13.19	12.57	6.89
5XE	Resistant	Inoculated	End of the season	Ambient	25.9	92131658	13911880358	80518352	73696904	79.99	13.91	12.61	8.47
6EM	Susceptible	Inoculated	Mid-season	Elevated ozone	39.8	83038324	12538786924	72028537	66836114	80.49	12.54	13.26	7.21
6EE	Susceptible	Inoculated	End of the season	Elevated ozone	41.8	85948910	12978285410	74964898	69762754	81.17	12.98	12.78	6.94
6XM	Resistant	Inoculated	Mid-season	Elevated ozone	25.3	72334912	10922571712	62846737	58011850	80.20	10.92	13.12	7.69
6XE	Resistant	Inoculated	End of the season	Elevated ozone	33.6	43692494	6597566594	38063184	29469316	67.45	6.60	12.88	22.58
7EE	Susceptible	Control	End of the season	Elevated ozone	36	80783162	12198257462	70281064	64307588	79.61	12.20	13.00	8.50
7XM	Resistant	Control	Mid-season	Elevated ozone	11.5	82013696	12384068096	73704235	69351830	84.56	12.38	10.13	5.91
7XE	Resistant	Control	End of the season	Elevated ozone	34.2	92715106	13999981006	82266875	74915476	80.80	14.00	11.27	8.94
8EE	Susceptible	Control	End of the season	Ambient	35.1	93149022	14065502322	82364507	76748744	82.39	14.07	11.58	6.82
8XM	Resistant	Control	Mid-season	Ambient	23.4	86912066	13123721966	75428445	69475752	79.94	13.12	13.21	7.89
8XE	Resistant	Control	End of the season	Ambient	33.3	87639550	13233572050	76683529	71598300	81.70	13.23	12.50	6.63
9EM	Susceptible	Control	Mid-season	Ambient	30.4	76739578	11587676278	66009506	60962488	79.44	11.59	13.98	7.65
9EE	Susceptible	Control	End of the season	Ambient	44.6	74029888	11178513088	64536900	59974132	81.01	11.18	12.82	7.07
9XM	Resistant	Control	Mid-season	Ambient	23.4	107540380	16238597380	92551600	84397566	78.48	16.24	13.94	8.81
9XE	Resistant	Control	End of the season	Ambient	35.8	83594546	12622776446	73142122	66505530	79.56	12.62	12.50	9.07
10EM	Susceptible	Control	Mid-season	Elevated ozone	19.2	62672036	9463477436	52745864	45571498	72.71	9.46	15.84	13.60
10EE	Susceptible	Control	End of the season	Elevated ozone	14	79082574	11941468674	70079799	65237834	82.49	11.94	11.38	6.91
10XM	Resistant	Control	Mid-season	Elevated ozone	21	74972812	11320894612	64473571	58494178	78.02	11.32	14.00	9.27
10XE	Resistant	Control	End of the season	Elevated ozone	17.5	98574260	14884713260	85562399	78476258	79.61	14.88	13.20	8.28
11EM	Susceptible	Control	Mid-season	Elevated ozone	29.8	120049118	18127416818	105338540	96783338	80.62	18.13	12.25	8.12
11EE	Susceptible	Control	End of the season	Elevated ozone	13.84	76087608	11489228808	67008309	56202158	73.87	11.49	11.93	16.13
11XM	Resistant	Control	Mid-season	Elevated ozone	16.06	108823052	16432280852	95722349	87635384	80.53	16.43	12.04	8.45
11XE	Resistant	Control	End of the season	Elevated ozone	16.77	76221844	11509498444	66056543	55582900	72.92	11.51	13.34	15.86
12EM	Susceptible	Control	Mid-season	Ambient	17.5	113646040	17160552040	99066065	91624154	80.62	17.16	12.83	7.51
12XM	Resistant	Control	Mid-season	Ambient	38	60168800	9085488800	52245447	48209842	80.12	9.09	13.17	7.72
12XE	Resistant	Control	End of the season	Ambient	29.6	105040296	15861084696	91675213	85177988	81.09	15.86	12.72	7.09
OEC	Susceptible	Control	Base	Green house	34.6	41926072	6330836872	34883887	26811796	63.95	6.33	16.80	23.14
OEI	Susceptible	Inoculated	Base	Green house	13.35	92472636	13963368036	76986190	54694930	59.15	13.96	16.75	28.95
OXC	Resistant	Control	Base	Green house	13.94	18879282	2850771582	15442554	11542140	61.14	2.85	18.20	25.26
OXI	Resistant	Inoculated	Base	Green house	16.11	61371554	9267104654	51009338	31065826	50.62	9.27	16.88	39.10

Table S3A: Pairwise comparison for bacterial Chao1 diversity index for resistant pepper. Significance levels for each treatment combination are indicated by *p < 0.05; **p < 0.01; ***p < 0.001.

Comparisons	Mean difference	lwr	upr	Adjusted *p*-value
Control sample, ambient environment, and end of the season vs. Control sample, ambient environment, and base time point	153	-26.281695	332.2816953	0.1298768
Control sample, ambient environment, and mid-season vs. Control sample, ambient environment, and base time point	177.333333	-1.948362	356.6150286	0.0537938
Control sample, elevated ozone, and end of the season vs. Control sample, ambient environment, and base time point	196.333333	17.051638	375.6150286	0.0260945
Control sample, elevated ozone, and mid-season vs. Control sample, ambient environment, and base time point	253.333333	74.051638	432.6150286	0.0028437
Inoculated sample, ambient environment, and base time point vs. Control sample, ambient environment, and base time point	218	-1.574337	437.5743368	0.0524734
Inoculated sample, ambient environment, and end of the season vs. Control sample, ambient environment, and base time point	127	-52.281695	306.2816953	0.3003286
Inoculated sample, ambient environment, and mid-season vs. Control sample, ambient environment, and base time point	193	13.718305	372.2816953	0.0296671
Inoculated sample, elevated ozone, and end of the season vs. Control sample, ambient environment, and base time point	139.666667	-39.615029	318.9483619	0.203141
Inoculated sample, elevated ozone, and mid-season vs. Control sample, ambient environment, and base time point	213.333333	34.051638	392.6150286	0.0134904
Control sample, ambient environment, and mid-season vs. Control sample, ambient environment, and end of the season	24.333333	-102.437969	151.1046358	0.9991099
Control sample, elevated ozone, and end of the season vs. Control sample, ambient environment, and end of the season	43.333333	-83.437969	170.1046358	0.9527894
Control sample, elevated ozone, and mid-season vs. Control sample, ambient environment, and end of the season	100.333333	-26.437969	227.1046358	0.1889544
Inoculated sample, ambient environment, and base time point vs. Control sample, ambient environment, and end of the season	65	-114.281695	244.2816953	0.9343351
Inoculated sample, ambient environment, and end of the season vs. Control sample, ambient environment, and end of the season	-26	-152.771302	100.7713025	0.9985192
Inoculated sample, ambient environment, and mid-season vs. Control sample, ambient environment, and end of the season	40	-86.771302	166.7713025	0.9706473
Inoculated sample, elevated ozone, and end of the season vs. Control sample, ambient environment, and end of the season	-13.333333	-140.104636	113.4379691	0.9999938
Inoculated sample, elevated ozone, and mid-season vs. Control sample, ambient environment, and end of the season	60.333333	-66.437969	187.1046358	0.7636049
Control sample, elevated ozone, and end of the season vs. Control sample, ambient environment, and mid-season	19	-107.771302	145.7713025	0.9998771
Control sample, elevated ozone, and mid-season vs. Control sample, ambient environment, and mid-season	76	-50.771302	202.7713025	0.5029492
Inoculated sample, ambient environment, and base time point vs. Control sample, ambient environment, and mid-season	40.666667	-138.615029	219.9483619	0.9968571
Inoculated sample, ambient environment, and end of the season vs. Control sample, ambient environment, and mid-season	-50.333333	-177.104636	76.4379691	0.8945303
Inoculated sample, ambient environment, and mid-season vs. Control sample, ambient environment, and mid-season	15.666667	-111.104636	142.4379691	0.9999754
Inoculated sample, elevated ozone, and end of the season vs. Control sample, ambient environment, and mid-season	-37.666667	-164.437969	89.1046358	0.9798423
Inoculated sample, elevated ozone, and mid-season vs. Control sample, ambient environment, and mid-season	36	-90.771302	162.7713025	0.9849613
Control sample, elevated ozone, and mid-season vs. Control sample, elevated ozone, and end of the season	57	-69.771302	183.7713025	0.8126418
Inoculated sample, ambient environment, and base time point vs. Control sample, elevated ozone, and end of the season	21.666667	-157.615029	200.9483619	0.9999796
Inoculated sample, ambient environment, and end of the season vs. Control sample, elevated ozone, and end of the season	-69.333333	-196.104636	57.4379691	0.615523
Inoculated sample, ambient environment, and mid-season vs. Control sample, elevated ozone, and end of the season	-3.333333	-130.104636	123.4379691	1
Inoculated sample, elevated ozone, and end of the season vs. Control sample, elevated ozone, and end of the season	-56.666667	-183.437969	70.1046358	0.8172832
Inoculated sample, elevated ozone, and mid-season vs. Control sample, elevated ozone, and end of the season	17	-109.771302	143.7713025	0.9999511
Inoculated sample, ambient environment, and base time point vs. Control sample, elevated ozone, and mid-season	-35.333333	-214.615029	143.9483619	0.9989083
Inoculated sample, ambient environment, and end of the season vs. Control sample, elevated ozone, and mid-season	-126.333333	-253.104636	0.4379691	0.0511777
Inoculated sample, ambient environment, and mid-season vs. Control sample, elevated ozone, and mid-season	-60.333333	-187.104636	66.4379691	0.7636049
Inoculated sample, elevated ozone, and end of the season vs. Control sample, elevated ozone, and mid-season	-113.666667	-240.437969	13.1046358	0.098838
Inoculated sample, elevated ozone, and mid-season vs. Control sample, elevated ozone, and mid-season	-40	-166.771302	86.7713025	0.9706473
Inoculated sample, ambient environment, and end of the season vs. Inoculated sample, ambient environment, and base time point	-91	-270.281695	88.2816953	0.6995067
Inoculated sample, ambient environment, and mid-season vs. Inoculated sample, ambient environment, and base time point	-25	-204.281695	154.2816953	0.9999323
Inoculated sample, elevated ozone, and end of the season vs. Inoculated sample, ambient environment, and base time point	-78.333333	-257.615029	100.9483619	0.8345678
Inoculated sample, elevated ozone, and mid-season vs. Inoculated sample, ambient environment, and base time point	-4.666667	-183.948362	174.6150286	1
Inoculated sample, ambient environment, and mid-season vs. Inoculated sample, ambient environment, and end of the season	66	-60.771302	192.7713025	0.6720025
Inoculated sample, elevated ozone, and end of the season vs. Inoculated sample, ambient environment, and end of the season	12.666667	-114.104636	139.4379691	0.999996
Inoculated sample, elevated ozone, and mid-season vs. Inoculated sample, ambient environment, and end of the season	86.333333	-40.437969	213.1046358	0.3456482
Inoculated sample, elevated ozone, and end of the season vs. Inoculated sample, ambient environment, and mid-season	-53.333333	-180.104636	73.4379691	0.8606658
Inoculated sample, elevated ozone, and mid-season vs. Inoculated sample, ambient environment, and mid-season	20.333333	-106.437969	147.1046358	0.9997862
Inoculated sample, elevated ozone, and mid-season vs. Inoculated sample, elevated ozone, and end of the season	73.666667	-53.104636	200.4379691	0.5418761

Table S3B: Pairwise comparison for bacterial Shanon diversity index(Dunn's multiple comparison test) for resistant pepper. Significance levels for each treatment combination are indicated by *p < 0.05; **p < 0.01; ***p < 0.001.

Comparisons	Z	Unadjusted *p*	*p* adjusted	Significance
Control sample, ambient environment, and base time point vs. Control sample, ambient environment, and end of the season	-1.13227703	0.257517982	0.57941546	
Control sample, ambient environment, and base time point vs. Control sample, ambient environment, and mid-season	-1.32098987	0.186504741	0.52454458	
Control sample, ambient environment, and end of the season vs. Control sample, ambient environment, and mid-season	-0.26688026	0.789561359	0.88825653	
Control sample, ambient environment, and base time point vs. Control sample, elevated ozone, and end of the season	-0.83033649	0.406348562	0.6772476	
Control sample, ambient environment, and end of the season vs. Control sample, elevated ozone, and end of the season	0.42700841	0.669373202	0.86062269	
Control sample, ambient environment, and mid-season vs. Control sample, elevated ozone, and end of the season	0.69388867	0.487752032	0.70802714	
Control sample, ambient environment, and base time point vs. Control sample, elevated ozone, and mid-season	-2.18906893	0.02859183	0.25732647	
Control sample, ambient environment, and end of the season vs. Control sample, elevated ozone, and mid-season	-1.49452944	0.135037294	0.46743679	
Control sample, ambient environment, and mid-season vs. Control sample, elevated ozone, and mid-season	-1.22764918	0.219578692	0.54894673	
Control sample, elevated ozone, and end of the season vs. Control sample, elevated ozone, and mid-season	-1.92153785	0.054663936	0.35141102	
Control sample, ambient environment, and base time point vs. Inoculated sample, ambient environment, and base time point	-1.01695036	0.309177045	0.63240759	
Control sample, ambient environment, and end of the season vs. Inoculated sample, ambient environment, and base time point	-0.1132277	0.909850033	0.95216864	
Control sample, ambient environment, and mid-season vs. Inoculated sample, ambient environment, and base time point	0.07548514	0.939828724	0.93982872	
Control sample, elevated ozone, and end of the season vs. Inoculated sample, ambient environment, and base time point	-0.41516825	0.678018743	0.82461739	
Control sample, elevated ozone, and mid-season vs. Inoculated sample, ambient environment, and base time point	0.9435642	0.345392396	0.62170631	
Control sample, ambient environment, and base time point vs. Inoculated sample, ambient environment, and end of the season	0.07548514	0.939828724	0.96118847	
Control sample, ambient environment, and end of the season vs. Inoculated sample, ambient environment, and end of the season	1.70803364	0.087630101	0.4381505	
Control sample, ambient environment, and mid-season vs. Inoculated sample, ambient environment, and end of the season	1.9749139	0.04827792	0.3620844	
Control sample, elevated ozone, and end of the season vs. Inoculated sample, ambient environment, and end of the season	1.28102523	0.200184804	0.52990095	
Control sample, elevated ozone, and mid-season vs. Inoculated sample, ambient environment, and end of the season	3.20256308	0.001362105	0.06129471	
Inoculated sample, ambient environment, and base time point vs. Inoculated sample, ambient environment, and end of the season	1.32098987	0.186504741	0.55951422	
Control sample, ambient environment, and base time point vs. Inoculated sample, ambient environment, and mid-season	-1.66067298	0.096779142	0.43550614	
Control sample, ambient environment, and end of the season vs. Inoculated sample, ambient environment, and mid-season	-0.74726472	0.454903785	0.73109537	
Control sample, ambient environment, and mid-season vs. Inoculated sample, ambient environment, and mid-season	-0.48038446	0.630954041	0.83508623	
Control sample, elevated ozone, and end of the season vs. Inoculated sample, ambient environment, and mid-season	-1.17427313	0.240285643	0.56909758	
Control sample, elevated ozone, and mid-season vs. Inoculated sample, ambient environment, and mid-season	0.74726472	0.454903785	0.70588518	
Inoculated sample, ambient environment, and base time point vs. Inoculated sample, ambient environment, and mid-season	-0.41516825	0.678018743	0.84752343	
Inoculated sample, ambient environment, and end of the season vs. Inoculated sample, ambient environment, and mid-season	-2.45529836	0.01407677	0.21115155	
Control sample, ambient environment, and base time point vs. Inoculated sample, elevated ozone, and end of the season	-0.33968311	0.734095182	0.86932324	
Control sample, ambient environment, and end of the season vs. Inoculated sample, elevated ozone, and end of the season	1.12089708	0.262331675	0.5621393	
Control sample, ambient environment, and mid-season vs. Inoculated sample, elevated ozone, and end of the season	1.38777733	0.165204859	0.53101562	
Control sample, elevated ozone, and end of the season vs. Inoculated sample, elevated ozone, and end of the season	0.69388867	0.487752032	0.73162805	
Control sample, elevated ozone, and mid-season vs. Inoculated sample, elevated ozone, and end of the season	2.61542651	0.00891161	0.20051122	
Inoculated sample, ambient environment, and base time point vs. Inoculated sample, elevated ozone, and end of the season	0.90582163	0.365030272	0.63178316	
Inoculated sample, ambient environment, and end of the season vs. Inoculated sample, elevated ozone, and end of the season	-0.58713656	0.557111993	0.78343874	
Inoculated sample, ambient environment, and mid-season vs. Inoculated sample, elevated ozone, and end of the season	1.86816179	0.061739522	0.34728481	
control sample, ambient environment, and base time point vs. Inoculated sample, elevated ozone, and mid-season	-1.50970271	0.131119299	0.49169737	
Control sample, ambient environment, and end of the season vs. Inoculated sample, elevated ozone, and mid-season	-0.53376051	0.593507237	0.80932805	
Control sample, ambient environment, and mid-season vs. Inoculated sample, elevated ozone, and mid-season	-0.26688026	0.789561359	0.91103234	
Control sample, elevated ozone, and end of the season vs. Inoculated sample, elevated ozone, and mid-season	-0.96076892	0.336668368	0.65869898	
Control sample, elevated ozone, and mid-season vs. Inoculated sample, elevated ozone, and mid-season	0.96076892	0.336668368	0.63125319	
Inoculated sample, ambient environment, and base time point vs. Inoculated sample, elevated ozone, and mid-season	-0.26419797	0.791627372	0.86885931	
Inoculated sample, ambient environment, and end of the season vs. Inoculated sample, elevated ozone, and mid-season	-2.24179415	0.024974679	0.28096514	
Inoculated sample, ambient environment, and mid-season vs. Inoculated sample, elevated ozone, and mid-season	0.21350421	0.83093371	0.89028612	
Inoculated sample, elevated ozone, and end of the season vs. Inoculated sample, elevated ozone, and mid-season	-1.65465759	0.097993975	0.40088444	

Table S3C: Pairwise comparison for bacterial Chao1 diversity index for susceptible pepper. Significance levels for each treatment combination are indicated by ****p* **< 0.05; *****p* **< 0.01; ******p* **< 0.001.

Comparisons	diff	lwr	upr	*p* adjusted	Significance
Control sample, ambient environment, and end of the season vs. Control sample, ambient environment, and base time point	128	-88.71689	344.716891	0.4882834	
Control sample, ambient environment, and mid-season vs. Control sample, ambient environment, and base time point	141.5	-75.21689	358.216891	0.3688167	
Control sample, elevated ozone, and end of the season vs. Control sample, ambient environment, and base time point	110.666667	-93.65598	314.989311	0.5917825	
Control sample, elevated ozone, and mid-season vs. Control sample, ambient environment, and base time point	232	15.28311	448.716891	0.0319968	
Inoculated sample, ambient environment, and base time point vs. Control sample, ambient environment, and base time point	236	-14.24311	486.243111	0.0713791	
Inoculated sample, ambient environment, and end of the season vs. Control sample, ambient environment, and base time point	-63.666667	-267.98931	140.655978	0.964853	
Inoculated sample, ambient environment, and mid-season vs. Control sample, ambient environment, and base time point	66.333333	-137.98931	270.655978	0.9554074	
Inoculated sample, elevated ozone, and end of the season vs. Control sample, ambient environment, and base time point	-62.333333	-266.65598	141.989311	0.9690089	
Inoculated sample, elevated ozone, and mid-season vs. Control sample, ambient environment, and base time point	27.333333	-176.98931	231.655978	0.9999279	
Control sample, ambient environment, and mid-season vs. Control sample, ambient environment, and end of the season	13.5	-163.4486	190.448601	0.9999994	
Control sample, elevated ozone, and end of the season vs. Control sample, ambient environment, and end of the season	-17.333333	-178.86457	144.1979	0.9999887	
Control sample, elevated ozone, and mid-season vs. Control sample, ambient environment, and end of the season	104	-72.9486	280.948601	0.4942194	
Inoculated sample, ambient environment, and base time point vs. Control sample, ambient environment, and end of the season	108	-108.71689	324.716891	0.6846657	
Inoculated sample, ambient environment, and end of the season vs. Control sample, ambient environment, and end of the season	-191.666667	-353.1979	-30.135433	0.015289	*
Inoculated sample, ambient environment, and mid-season vs. Control sample, ambient environment, and end of the season	-61.666667	-223.1979	99.864567	0.894788	
Inoculated sample, elevated ozone, and end of the season vs. Control sample, ambient environment, and end of the season	-190.333333	-351.86457	-28.8021	0.0161128	*
Inoculated sample, elevated ozone, and mid-season vs. Control sample, ambient environment, and end of the season	-100.666667	-262.1979	60.864567	0.4236153	
Control sample, elevated ozone, and end of the season vs. Control sample, ambient environment, and mid-season	-30.833333	-192.36457	130.6979	0.998772	
Control sample, elevated ozone, and mid-season vs. Control sample, ambient environment, and mid-season	90.5	-86.4486	267.448601	0.6567579	
Inoculated sample, ambient environment, and base time point vs. Control sample, ambient environment, and mid-season	94.5	-122.21689	311.216891	0.8085275	
Inoculated sample, ambient environment, and end of the season vs. Control sample, ambient environment, and mid-season	-205.166667	-366.6979	-43.635433	0.0090049	**
Inoculated sample, ambient environment, and mid-season vs. Control sample, ambient environment, and mid-season	-75.166667	-236.6979	86.364567	0.7526888	
Inoculated sample, elevated ozone, and end of the season vs. Control sample, ambient environment, and mid-season	-203.833333	-365.36457	-42.3021	0.0094863	**
Inoculated sample, elevated ozone, and mid-season vs. Control sample, ambient environment, and mid-season	-114.166667	-275.6979	47.364567	0.2815983	
Control sample, elevated ozone, and mid-season vs. Control sample, elevated ozone, and end of the season	121.333333	-40.1979	282.864567	0.2220085	
Inoculated sample, ambient environment, and base time point vs. Control sample, elevated ozone, and end of the season	125.333333	-78.98931	329.655978	0.4425976	
Inoculated sample, ambient environment, and end of the season vs. Control sample, elevated ozone, and end of the season	-174.333333	-318.81126	-29.855406	0.0134579	*
Inoculated sample, ambient environment, and mid-season vs. Control sample, elevated ozone, and end of the season	-44.333333	-188.81126	100.144594	0.9679123	
Inoculated sample, elevated ozone, and end of the season vs. Control sample, elevated ozone, and end of the season	-173	-317.47793	-28.522073	0.0142701	*
Inoculated sample, elevated ozone, and mid-season vs. Control sample, elevated ozone, and end of the season	-83.333333	-227.81126	61.144594	0.5169461	
Inoculated sample, ambient environment, and base time point vs. Control sample, elevated ozone, and mid-season	4	-212.71689	220.716891	1	
Inoculated sample, ambient environment, and end of the season vs. Control sample, elevated ozone, and mid-season	-295.666667	-457.1979	-134.135433	0.000322	*
Inoculated sample, ambient environment, and mid-season vs. Control sample, elevated ozone, and mid-season	-165.666667	-327.1979	-4.135433	0.042541	*
Inoculated sample, elevated ozone, and end of the season vs. Control sample, elevated ozone, and mid-season	-294.333333	-455.86457	-132.8021	0.0003371	***
Inoculated sample, elevated ozone, and mid-season vs. Control sample, elevated ozone, and mid-season	-204.666667	-366.1979	-43.135433	0.0091824	***
Inoculated sample, ambient environment, and end of the season vs. Inoculated sample, ambient environment, and base time point	-299.666667	-503.98931	-95.344022	0.0026616	**
Inoculated sample, ambient environment, and mid-season vs. Inoculated sample, ambient environment, and base time point	-169.666667	-373.98931	34.655978	0.1413235	
Inoculated sample, elevated ozone, and end of the season vs. Inoculated sample, ambient environment, and base time point	-298.333333	-502.65598	-94.010689	0.0027695	**
Inoculated sample, elevated ozone, and mid-season vs. Inoculated sample, ambient environment, and base time point	-208.666667	-412.98931	-4.344022	0.0437245	*
Inoculated sample, ambient environment, and mid-season vs. Inoculated sample, ambient environment, and end of the season	130	-14.47793	274.477927	0.0931871	*
Inoculated sample, elevated ozone, and end of the season vs. Inoculated sample, ambient environment, and end of the season	1.333333	-143.14459	145.811261	1	
Inoculated sample, elevated ozone, and mid-season vs. Inoculated sample, ambient environment, and end of the season	91	-53.47793	235.477927	0.4109739	
Inoculated sample, elevated ozone, and end of the season vs. Inoculated sample, ambient environment, and mid-season	-128.666667	-273.14459	15.811261	0.0985778	
Inoculated sample, elevated ozone, and mid-season vs. Inoculated sample, ambient environment, and mid-season	-39	-183.47793	105.477927	0.9855907	
Inoculated sample, elevated ozone, and mid-season vs. Inoculated sample, elevated ozone, and end of the season	89.666667	-54.81126	234.144594	0.4285686	

Table S3D: Pairwise comparison for bacterial Shanon diversity index (Kruskal-Wallis multiple comparisons & *p*-values adjusted with the Benjamini-Hochberg method) for control susceptible pepper

Comparisons	Z	*p* unadjusted	*p* adjusted	Significance
Control sample, elevated ozone, and end of the season vs. Inoculated sample, elevated ozone, and end of the season	2.4618298	0.01382302	0.04146907	*
Control sample, elevated ozone, and end of the season vs. Control sample, elevated ozone, and mid-season	-0.2752409	0.78313113	0.78313113	
Control sample, elevated ozone, and mid-season vs. Inoculated sample, elevated ozone, and end of the season	2.4771685	0.01324294	0.07945763	
Control sample, elevated ozone, and end of the season vs. Inoculated sample, elevated ozone, and mid-season	1.3540064	0.17573434	0.2636015	
Control sample, elevated ozone, and mid-season vs. Inoculated sample, elevated ozone, and mid-season	1.4863011	0.1371995	0.274399	
Inoculated sample, elevated ozone, and end of the season vs. Inoculated sample, elevated ozone, and mid-season	-1.1078234	0.26793808	0.3215257	

Table S3E: Pairwise comparison for bacterial Shanon diversity index for inoculated susceptible pepper. Significance levels of treatment combination are indicated by *p < 0.05; **p < 0.01; ***p < 0.001.

Comparisons	diff	lwr	upr	*p* adjusted	Significance
Inoculated sample, ambient environment, and end of the season vs. Control sample, ambient environment, and end of the season	-2.2891591	-3.3399312	-1.238387	0.0011631	**
Inoculated sample, ambient environment, and mid-season vs. Control sample, ambient environment, and end of the season	-1.6760916	-2.7268637	-0.6253195	0.0059681	**
Inoculated sample, ambient environment, and end of the season vs. Control sample, ambient environment, and mid-season	-3.080265	-4.1310371	-2.0294929	0.000223	***
Inoculated sample, ambient environment, and mid-season vs. Control sample, ambient environment, and mid-season	-2.4671976	-3.5179697	-1.4164255	0.000772	***
Control sample, ambient environment, and mid-season vs. Control sample, ambient environment, and end of the season	0.7911059	-0.3599572	1.9421691	0.1817845	
Inoculated sample, ambient environment, and mid-season vs. Inoculated sample, ambient environment, and end of the season	0.6130674	-0.3267717	1.5529066	0.2101921	

Table S4A: Pairwise comparison of Eukaryotic Chao1 diversity index for resistant pepper. Significance levels for each treatment combination are indicated by ****p* **< 0.05; *****p* **< 0.01; ******p* **< 0.001.

**Comparisons**	**diff**	**lwr**	**upr**	***p* adjusted**	**Significance**
Control, ambient environment, and mid-season vs. Control, ambient environment, and end of the season	7.3333333	-2.5464459	17.213113	0.231451	
Control, elevated ozone, and end of the season vs. Control, ambient environment, and end of the season	2	-7.8797792	11.879779	0.995412	
Control, elevated ozone, and mid-season vs. Control, ambient environment, and end of the season	10	0.1202208	19.879779	0.0462689	*
Inoculated, ambient environment, and end of the season vs. Control, ambient environment, and end of the season	2.6666667	-7.2131125	12.546446	0.9761333	
Inoculated, ambient environment, and mid-season vs. Control, ambient environment, and end of the season	3.6666667	-7.3792623	14.712596	0.93174	
Inoculated, elevated ozone, and end of the season vs. Control, ambient environment, and end of the season	2.3333333	-7.5464459	12.213113	0.988641	
Inoculated, elevated ozone, and mid-season vs. Control, ambient environment, and end of the season	6.3333333	-3.5464459	16.213113	0.383007	
Control, elevated ozone, and end of the season vs. Control, ambient environment, and mid-season	-5.3333333	-15.2131125	4.546446	0.578869	
Control, elevated ozone, and mid-season vs. Control, ambient environment, and mid-season	2.6666667	-7.2131125	12.546446	0.9761333	
Inoculated, ambient environment, and end of the season vs. Control, ambient environment, and mid-season	-4.6666667	-14.5464459	5.213113	0.716115	
Inoculated, ambient environment, and mid-season vs. Control, ambient environment, and mid-season	-3.6666667	-14.7125956	7.379262	0.93174	
Inoculated, elevated ozone, and end of the season vs. Control, ambient environment, and mid-season	-5	-14.8797792	4.879779	0.6482302	
Inoculated, elevated ozone, and mid-season vs. Control, ambient environment, and mid-season	-1	-10.8797792	8.879779	0.9999477	
Control, elevated ozone, and mid-season vs. Control, elevated ozone, and end of the season	8	-1.8797792	17.879779	0.1592666	
Inoculated, ambient environment, and end of the season vs. Control, elevated ozone, and end of the season	0.6666667	-9.2131125	10.546446	0.9999967	
Inoculated, ambient environment, and mid-season vs. Control, elevated ozone, and end of the season	1.6666667	-9.3792623	12.712596	0.9992691	
Inoculated, elevated ozone, and end of the season vs. Control, elevated ozone, and end of the season	0.3333333	-9.5464459	10.213113	1	
Inoculated, elevated ozone, and mid-season vs. Control, elevated ozone, and end of the season	4.3333333	-5.5464459	14.213113	0.7800355	
Inoculated, ambient environment, and end of the season vs. Control, elevated ozone, and mid-season	-7.3333333	-17.2131125	2.546446	0.231451	
Inoculated, ambient environment, and mid-season vs. Control, elevated ozone, and mid-season	-6.3333333	-17.3792623	4.712596	0.5107304	
Inoculated, elevated ozone, and end of the season vs. Control, elevated ozone, and mid-season	-7.6666667	-17.5464459	2.213113	0.1926158	
Inoculated, elevated ozone, and mid-season vs. Control, elevated ozone, and mid-season	-3.6666667	-13.5464459	6.213113	0.8867681	
Inoculated, ambient environment, and mid-season vs. Inoculated, ambient environment, and end of the season	1	-10.0459289	12.045929	0.9999754	
Inoculated, elevated ozone, and end of the season vs. Inoculated, ambient environment, and end of the season	-0.3333333	-10.2131125	9.546446	1	
Inoculated, elevated ozone, and mid-season vs. Inoculated, ambient environment, and end of the season	3.6666667	-6.2131125	13.546446	0.8867681	
Inoculated, elevated ozone, and end of the season vs. Inoculated, ambient environment, and mid-season	-1.3333333	-12.3792623	9.712596	0.9998302	
Inoculated, elevated ozone, and mid-season vs. Inoculated, ambient environment, and mid-season	2.6666667	-8.3792623	13.712596	0.9871275	
Inoculated, elevated ozone, and mid-season vs. Inoculated, elevated ozone, and end of the season	4	-5.8797792	13.879779	0.8375864	

Table S4B: Pairwise comparison of Eukaryotic Shannon diversity index for resistant pepper. Significance levels for each treatment combination are indicated by ****p* **< 0.05; *****p* **< 0.01; ******p* **< 0.001.

Comparisons	diff	lwr	upr	*p* adjusted	Significance
Control, ambient environment, and mid-season vs. Control, ambient environment, and end of the season	0.20217503	-0.5332479	0.93759798	0.9736648	
Control, elevated ozone, and end of the season vs. Control, ambient environment, and end of the season	0.060349817	-0.6750731	0.79577277	0.9999874	
Control, elevated ozone, and mid-season vs. Control, ambient environment, and end of the season	-0.098997082	-0.83442	0.63642587	0.9996517	
Inoculated, ambient environment, and end of the season vs. Control, ambient environment, and end of the season	0.26322271	-0.4722002	0.99864566	0.9034352	
Inoculated, ambient environment, and mid-season vs. Control, ambient environment, and end of the season	0.262638007	-0.5595899	1.08486587	0.9432662	
Inoculated, elevated ozone, and end of the season vs. Control, ambient environment, and end of the season	0.168106746	-0.5673162	0.9035297	0.9905876	
Inoculated, elevated ozone, and mid-season vs. Control, ambient environment, and end of the season	-0.394374595	-1.1297975	0.34104836	0.5862019	
Control, elevated ozone, and end of the season vs. Control, ambient environment, and mid-season	-0.141825214	-0.8772482	0.59359774	0.9965823	
Control, elevated ozone, and mid-season vs. Control, ambient environment, and mid-season	-0.301172112	-1.0365951	0.43425084	0.830105	
Inoculated, ambient environment, and end of the season vs. Control, ambient environment, and mid-season	0.06104768	-0.6743753	0.79647063	0.9999864	
Inoculated, ambient environment, and mid-season vs. Control, ambient environment, and mid-season	0.060462976	-0.7617649	0.88269083	0.9999941	
Inoculated, elevated ozone, and end of the season vs. Control, ambient environment, and mid-season	-0.034068284	-0.7694912	0.70135467	0.9999998	
Inoculated, elevated ozone, and mid-season vs. Control, ambient environment, and mid-season	-0.596549625	-1.3319726	0.13887333	0.1579671	
Control, elevated ozone, and mid-season vs. Control, elevated ozone, and end of the season	-0.159346899	-0.8947699	0.57607606	0.9931169	
Inoculated, ambient environment, and end of the season vs. Control, elevated ozone, and end of the season	0.202872893	-0.5325501	0.93829585	0.9731763	
Inoculated, ambient environment, and mid-season vs. Control, elevated ozone, and end of the season	0.20228819	-0.6199397	1.02451605	0.9856766	
Inoculated, elevated ozone, and end of the season vs. Control, elevated ozone, and end of the season	0.107756929	-0.627666	0.84317988	0.9993951	
Inoculated, elevated ozone, and mid-season vs. Control, elevated ozone, and end of the season	-0.454724412	-1.1901474	0.28069854	0.4239381	
Inoculated, ambient environment, and end of the season vs. Control, elevated ozone, and mid-season	0.362219792	-0.3732032	1.09764275	0.6758122	
Inoculated, ambient environment, and mid-season vs. Control, elevated ozone, and mid-season	0.361635089	-0.4605928	1.18386295	0.7778266	
Inoculated, elevated ozone, and end of the season vs. Control, elevated ozone, and mid-season	0.267103828	-0.4683191	1.00252678	0.8969574	
Inoculated, elevated ozone, and mid-season vs. Control, elevated ozone, and mid-season	-0.295377513	-1.0308005	0.44004544	0.8426747	
Inoculated, ambient environment, and mid-season vs. Inoculated, ambient environment, and end of the season	-0.000584704	-0.8228126	0.82164315	1	
Inoculated, elevated ozone, and end of the season vs. Inoculated, ambient environment, and end of the season	-0.095115964	-0.8305389	0.64030699	0.9997321	
Inoculated, elevated ozone, and mid-season vs. Inoculated, ambient environment, and end of the season	-0.657597305	-1.3930203	0.07782565	0.0966907	
Inoculated, elevated ozone, and end of the season vs. Inoculated, ambient environment, and mid-season	-0.094531261	-0.9167591	0.7276966	0.9998772	
Inoculated, elevated ozone, and mid-season vs. Inoculated, ambient environment, and mid-season	-0.657012601	-1.4792405	0.16521526	0.1692467	
Inoculated, elevated ozone, and mid-season vs. Inoculated, elevated ozone, and end of the season	-0.562481341	-1.2979043	0.17294161	0.2048296	

Table S4C: Pairwise comparison of Eukaryotic Chao1 diversity index for susceptible pepper. Significance levels for each treatment combination are indicated by *p < 0.05; **p < 0.01; ***p < 0.001; ****p < 0.0001.

Comparisons	diff	lwr	upr	p adjusted	Significance
Control, ambient environment, and mid-season vs. Control, ambient environment, and end of the season	13	-4.108058	30.1080581	0.2015098	
Control, elevated ozone, and end of the season vs. Control, ambient environment, and end of the season	8.333333	-7.284116	23.9507822	0.5700742	
Control, elevated ozone, and mid-season vs. Control, ambient environment, and end of the season	19.5	2.391942	36.6080581	0.021199	*
Inoculated, ambient environment, and end of the season vs. Control, ambient environment, and end of the season	-13.333333	-28.950782	2.2841156	0.1193093	
Inoculated, ambient environment, and mid-season vs. Control, ambient environment, and end of the season	-4	-19.617449	11.6174489	0.9790401	
Inoculated, elevated ozone, and end of the season vs. Control, ambient environment, and end of the season	-14.666667	-30.284116	0.9507822	0.072205	
Inoculated, elevated ozone, and mid-season vs. Control, ambient environment, and end of the season	-5	-20.617449	10.6174489	0.9347312	
Control, elevated ozone, and end of the season vs. Control, ambient environment, and mid-season	-4.666667	-20.284116	10.9507822	0.9532884	
Control, elevated ozone, and mid-season vs. Control, ambient environment, and mid-season	6.5	-10.608058	23.6080581	0.8610813	
Inoculated, ambient environment, and end of the season vs. Control, ambient environment, and mid-season	-26.333333	-41.950782	-10.7158844	0.0008164	***
Inoculated, ambient environment, and mid-season vs. Control, ambient environment, and mid-season	-17	-32.617449	-1.3825511	0.0290765	*
Inoculated, elevated ozone, and end of the season vs. Control, ambient environment, and mid-season	-27.666667	-43.284116	-12.0492178	0.0005076	***
Inoculated, elevated ozone, and mid-season vs. Control, ambient environment, and mid-season	-18	-33.617449	-2.3825511	0.0195972	*
Control, elevated ozone, and mid-season vs. Control, elevated ozone, and end of the season	11.166667	-4.450782	26.7841156	0.2550805	
Inoculated, ambient environment, and end of the season vs. Control, elevated ozone, and end of the season	-21.666667	-35.635338	-7.6979957	0.0017689	**
Inoculated, ambient environment, and mid-season vs. Control, elevated ozone, and end of the season	-12.333333	-26.302004	1.6353376	0.1007084	
Inoculated, elevated ozone, and end of the season vs. Control, elevated ozone, and end of the season	-23	-36.968671	-9.031329	0.0010214	**
Inoculated, elevated ozone, and mid-season vs. Control, elevated ozone, and end of the season	-13.333333	-27.302004	0.6353376	0.0658351	
Inoculated, ambient environment, and end of the season vs. Control, elevated ozone, and mid-season	-32.833333	-48.450782	-17.2158844	0.0000893	****
Inoculated, ambient environment, and mid-season vs. Control, elevated ozone, and mid-season	-23.5	-39.117449	-7.8825511	0.0023201	**
Inoculated, elevated ozone, and end of the season vs. Control, elevated ozone, and mid-season	-34.166667	-49.784116	-18.5492178	0.0000585	****
Inoculated, elevated ozone, and mid-season vs. Control, elevated ozone, and mid-season	-24.5	-40.117449	-8.8825511	0.0015964	**
Inoculated, ambient environment, and mid-season vs. Inoculated, ambient environment, and end of the season	9.333333	-4.635338	23.3020043	0.3220228	
Inoculated, elevated ozone, and end of the season vs. Inoculated, ambient environment, and end of the season	-1.333333	-15.302004	12.6353376	0.9999565	
Inoculated, elevated ozone, and mid-season vs. Inoculated, ambient environment, and end of the season	8.333333	-5.635338	22.3020043	0.445405	
Inoculated, elevated ozone, and end of the season vs. Inoculated, ambient environment, and mid-season	-10.666667	-24.635338	3.3020043	0.1974943	
Inoculated, elevated ozone, and mid-season vs. Inoculated, ambient environment, and mid-season	-1	-14.968671	12.968671	0.9999939	
Inoculated, elevated ozone, and mid-season vs. Inoculated, elevated ozone, and end of the season	9.666667	-4.302004	23.6353376	0.2865034	

Table S4D: Pairwise comparison of Eukaryotic Shanon diversity index for susceptible pepper. Significance levels for each treatment combination are indicated by *$p < 0.05$; **$p < 0.01$; ***$p < 0.001$.

Comparisons	diff	lwr	upr	*p* adjusted	Significance
Control, ambient environment, and mid-season vs. Control, ambient environment, and end of the season	-0.044112784	-0.9977797	0.90955417	0.9999997	
Control, elevated ozone, and end of the season vs. Control, ambient environment, and end of the season	-0.070985068	-0.9415599	0.79958977	0.9999851	
Control, elevated ozone, and mid-season vs. Control, ambient environment, and end of the season	-0.043791967	-0.9974589	0.90987498	0.9999997	
Inoculated, ambient environment, and end of the season vs. Control, ambient environment, and end of the season	-1.506356533	-2.3769314	-0.6357817	0.0006376	***
Inoculated, ambient environment, and mid-season vs. Control, ambient environment, and end of the season	-0.837815566	-1.7083904	0.03275927	0.0627951	
Inoculated, elevated ozone, and end of the season vs. Control, ambient environment, and end of the season	-1.543782445	-2.4143573	-0.67320761	0.0005027	***
Inoculated, elevated ozone, and mid-season vs. Control, ambient environment, and end of the season	-0.971433688	-1.8420085	-0.10085885	0.0245729	*
Control, elevated ozone, and end of the season vs. Control, ambient environment, and mid-season	-0.026872285	-0.8974471	0.84370255	1	
Control, elevated ozone, and mid-season vs. Control, ambient environment, and mid-season	0.000320816	-0.9533461	0.95398777	1	
Inoculated, ambient environment, and end of the season vs. Control, ambient environment, and mid-season	-1.462243749	-2.3328186	-0.59166891	0.0008469	***
Inoculated, ambient environment, and mid-season vs. Control, ambient environment, and mid-season	-0.793702783	-1.6642776	0.07687205	0.0850128	
Inoculated, elevated ozone, and end of the season vs. Control, ambient environment, and mid-season	-1.499669661	-2.3702445	-0.62909483	0.0006655	***
Inoculated, elevated ozone, and mid-season vs. Control, ambient environment, and mid-season	-0.927320904	-1.7978957	-0.05674607	0.0335637	*
Control, elevated ozone, and mid-season vs. Control, elevated ozone, and end of the season	0.027193101	-0.8433817	0.89776794	1	
Inoculated, ambient environment, and end of the season vs. Control, elevated ozone, and end of the season	-1.435371465	-2.2140373	-0.65670566	0.0003432	***
Inoculated, ambient environment, and mid-season vs. Control, elevated ozone, and end of the season	-0.766830498	-1.5454963	0.01183531	0.0548337	
Inoculated, elevated ozone, and end of the season vs. Control, elevated ozone, and end of the season	-1.472797377	-2.2514632	-0.69413157	0.0002653	***
Inoculated, elevated ozone, and mid-season vs. Control, elevated ozone, and end of the season	-0.900448619	-1.6791144	-0.12178282	0.0191385	*
Inoculated, ambient environment, and end of the season vs. Control, elevated ozone, and mid-season	-1.462564566	-2.3331394	-0.59198973	0.0008451	***
Inoculated, ambient environment, and mid-season vs. Control, elevated ozone, and mid-season	-0.794023599	-1.6645984	0.07655124	0.0848274	
Inoculated, elevated ozone, and end of the season vs. Control, elevated ozone, and mid-season	-1.499990478	-2.3705653	-0.62941564	0.0006641	***
Inoculated, elevated ozone, and mid-season vs. Control, elevated ozone, and mid-season	-0.92764172	-1.7982166	-0.05706689	0.0334878	*
Inoculated, ambient environment, and mid-season vs. Inoculated, ambient environment, and end of the season	0.668540967	-0.1101248	1.44720677	0.1160321	
Inoculated, elevated ozone, and end of the season vs. Inoculated, ambient environment, and end of the season	-0.037425912	-0.8160917	0.74123989	0.9999996	
Inoculated, elevated ozone, and mid-season vs. Inoculated, ambient environment, and end of the season	0.534922846	-0.243743	1.31358865	0.2937759	
Inoculated, elevated ozone, and end of the season vs. Inoculated, ambient environment, and mid-season	-0.705966879	-1.4846327	0.07269892	0.0875989	
Inoculated, elevated ozone, and mid-season vs. Inoculated, ambient environment, and mid-season	-0.133618121	-0.9122839	0.64504768	0.9979966	
Inoculated, elevated ozone, and mid-season vs. Inoculated, elevated ozone, and end of the season	0.572348758	-0.206317	1.35101456	0.2299224	

Table S5A: Permutational multivariant analysis of variance (PERMANOVA) of bacterial community composition based on Bray–Curtis dissimilarities of their relative abundance. Significance levels for each treatment combination are indicated by *$P < 0.05$; **$P < 0.01$; ***$P < 0.001$.

Variable	Df	Sum of sq	Mean Sqs	F.Model	R2	Pr(>F)	Significance
Cultivar	1	1.5289	1.5289	13.6497	0.12077	0.000999	***
Time of sampling	1	1.2936	1.2936	11.5487	0.10218	0.000999	***
Inoculation status	1	3.2369	3.2369	28.8983	0.25568	0.000999	***
Environment	1	0.3373	0.3373	3.0112	0.02664	0.023976	*
Cultivar:Time of sampling	1	0.2654	0.2654	2.3694	0.02096	0.052947	***
Cultivar:Inoculation status	1	0.9932	0.9932	8.8667	0.07845	0.000999	***
Time of sampling:Inoculation status	1	0.5124	0.5124	4.5742	0.04047	0.008991	**
Cultivar:Environment	1	0.1136	0.1136	1.0142	0.00897	0.330669	
Time of sampling:Environment	1	0.1164	0.1164	1.0389	0.00919	0.336663	
Inoculation status:Environment	1	0.1682	0.1682	1.5015	0.01328	0.172827	
Cultivar:Time of sampling:Inoculation status	1	0.3345	0.3345	2.986	0.02642	0.032967	*
Cultivar:Time of sampling:Environment	1	0.1147	0.1147	1.0242	0.00906	0.333666	
Cultivar:Inoculation status:Environment	1	0.0735	0.0735	0.6565	0.00581	0.632368	
Time of sampling:Inoculation status:Environment	1	0.1436	0.1436	1.2823	0.01135	0.241758	
Cultivar:Time of sampling:Inoculation status:Environment	1	0.1796	0.1796	1.6034	0.01419	0.16983	
Residuals	29	3.2483	0.112		0.25658		
Total	48	14.31			1		

Table S5B: Permutational multivariant analysis of variance (PERMANOVA) of bacterial community composition based on Bray–Curtis dissimilarities of relative abundance during the mid-season. Significance levels for each treatment combination are indicated by *$p < 0.05$; **$p < 0.01$; ***$p < 0.001$.

Variable	Df	Sum of sq	Mean Sqs	F.Model	R2	Pr(>F)	Significance
Cultivar	1	1.1685	1.16851	11.9388	0.23261	0.000999	***
Environment	1	0.2725	0.27248	2.784	0.05424	0.048951	*
Inoculation status	1	1.0519	1.05187	10.7471	0.20939	0.000999	***
Cultivar:Environment	1	0.0731	0.07313	0.7472	0.01456	0.515485	
Cultivar:Inoculation status	1	0.7896	0.78957	8.0671	0.15717	0.001998	**
Environment:Inoculation status	1	0.1676	0.16764	1.7127	0.03337	0.143856	
Cultivar:Inoculation status:Environment	1	0.1301	0.13012	1.3294	0.0259	0.208791	
Residuals	14	1.3703	0.09788		0.27276		
Total	21	5.0236			1		

Table S5C: Permutational multivariant analysis of variance (PERMANOVA) of bacterial community composition based on Bray–Curtis dissimilarities of relative abundance during end of the season. Significance levels for each treatment combination are indicated by *$p < 0.05$; **$p < 0.01$; ***$p < 0.001$.

Variable	Df	Sum of sq	Mean Sqs	F.Model	R2	Pr(>F)	Significance
Cultivar	1	0.3615	0.3615	4.038	0.06432	0.032967	*
Environment	1	0.1627	0.16272	1.818	0.02895	0.152847	
Inoculation status	1	3.1114	3.1114	34.755	0.55363	0.000999	***
Cultivar:Environment	1	0.1342	0.13425	1.5	0.02389	0.190809	
Cultivar:Inoculation status	1	0.2961	0.29615	3.308	0.05269	0.042957	*
Environment:Inoculation status	1	0.0881	0.08808	0.984	0.01567	0.336663	
Cultivar:Inoculation status:Environment	1	0.1231	0.12309	1.375	0.0219	0.223776	
Residuals	15	1.3429	0.08952		0.23894		
Total	22	5.62			1		

Table S5D: Permutational multivariant analysis of variance (PERMANOVA) of bacterial community composition based on Bray–Curtis dissimilarities of relative abundance in control samples of the resistant cultivar. Significance levels for each treatment combination are indicated by **p* < 0.05; ***p* < 0.01; ****p* < 0.001.

Variable	Df	Sum of sq	Mean Sqs	F.Model	R2	Pr(>F)	Significance
Environment	1	0.31574	0.31574	2.3301	0.1417	0.027	*
Time of sampling	1	0.64875	0.64875	4.7876	0.29115	0.001	***
Environment:Time of sampling	1	0.17973	0.17973	1.3264	0.08066	0.228	
Residuals	8	1.08405	0.13551		0.4865		
Total	11	2.22828			1		

Table S5E: Permutational multivariant analysis of variance (PERMANOVA) of bacterial community composition based on Bray–Curtis dissimilarities of relative abundance in samples from the elevated ozone. Significance levels for each treatment combination are indicated by *p < 0.05; **p < 0.01; ***p < 0.001.

Variable	Df	Sum of sq	Mean Sqs	F.Model	R2	Pr(>F)	Significance
Inoculation status	1	2.0005	2.00048	10.659	0.32704	0.001998	**
Chamber	4	0.5767	0.14417	0.7682	0.09428	0.691309	
Time of Sampling	1	0.7791	0.77909	4.1512	0.12737	0.015984	*
Residuals	11	2.0645	0.0912				
Total	22	6.117	0.18768				

Table S5F: Permutational multivariant analysis of variance (PERMANOVA) of bacterial community composition based on Bray–Curtis dissimilarities of relative abundance in samples from the ambient environment. Significance levels for each treatment combination are indicated by *p < 0.05; **p < 0.01; ***p < 0.001.

Variable	Df	Sum of sq	Mean Sqs	F.Model	R2	Pr(>F)	Significance
Inoculation status	1	2.06467	2.06467	10.9547	0.36955	0.000999	***
Chamber	4	0.4095	0.10239	0.5432	0.0733	0.856144	
Time of Sampling	1	0.7042	0.70423	3.7365	0.12605	0.026973	*
Residuals	10	1.8847	0.07308				
Total	21	5.5869	0.18847				

Table S6A: Permutational multivariant analysis of variance (PERMANOVA) of eukaryotes community composition based on Bray–Curtis dissimilarities of relative abundance. Significance levels for each treatment combination are indicated by *$p < 0.05$; **$p < 0.01$; ***$p < 0.001$.

Variable	Df	Sum of sq	R2	F	Pr(>F)	Significance
Time	1	1.8147	0.16834	9.3572	0.001	***
Environment	1	0.8229	0.07634	4.243	0.002	**
Time:Environment	1	0.3847	0.03569	1.9837	0.06	
Residual	40	7.7575	0.71963			
Total	43	10.7799	1			

Table S6B: Analysis of similarity (ANOSIM) of eukaryotes community composition based on Bray–Curtis dissimilarities of relative abundance influenced by different factors. Significance levels for each treatment combination are indicated by *$p < 0.05$; **$p < 0.01$; ***$p < 0.001$.

Variable	R-Value	P-Value	Significance
Susceptible vs Resistant	0.2092	0.001	**
Inoculation vs Control	0.1189	0.002	**

Table S6C: Permutational multivariant analysis of variance (PERMANOVA) of eukaryote community composition based on Bray–Curtis dissimilarities of relative abundance during mid-season. Significance levels for each treatment combination are indicated by *$p < 0.05$; **$p < 0.01$; ***$p < 0.001$.

Variable	Df	Sum of sq	R2	F	Pr(>F)	Significance
Cultivar	1	0.4475	0.1208	5.68	0.007	**
Environment	1	0.8238	0.22238	10.4565	0.001	***
Inoculation	1	0.5713	0.15421	7.2508	0.003	**
Cultivar:Environment	1	0.1401	0.03781	1.7779	0.134	
Cultivar:Inoculation	1	0.3258	0.08795	4.1352	0.012	*
Environment:inoculation	1	0.2071	0.05591	2.629	0.062	
Cultivar:Environment:Inoculation	1	0.1647	0.04447	2.0909	0.098	
Residual	13	1.0242	0.27648			
Total	20	3.7046	1			

Table S6D: Permutational multivariant analysis of variance (PERMANOVA) of eukaryote community composition based on Bray–Curtis dissimilarities of relative abundance during the end season. Significance levels for each treatment combination are indicated by *$p < 0.05$; **$p < 0.01$; ***$p < 0.001$.

Variable	Df	Sum of sq	R2	F	Pr(>F)	Significance
Environment	1	0.3513	0.06679	1.5029	0.19	
Residual	21	4.9092	0.93321			
Total	22	5.2606	1			

Table S6E: Analysis of similarity (ANOSIM) of eukaryotes community composition based on Bray–Curtis dissimilarities of relative abundance during end season. Significance levels for each treatment combination are indicated by *$p < 0.05$; **$p < 0.01$; ***$p < 0.001$.

Variable	R-Value	P-Value	Significance
Susceptible vs. Resistant	0.3747	0.001	**
Inoculation vs. Control	0.1157	0.035	*

Table S7A: Permutational multivariant analysis of variance (PERMANOVA) microbiota density across samples during mid-season and ambient environment based on Bray–Curtis dissimilarities in Control samples. Significance levels for each treatment combination are indicated by *p < 0.05; **p < 0.01; ***p < 0.001.

Variable	Df	Sum of sq	R2	F	Pr(>F)	Significance
Treatments	3	0.03898	0.01299	1.012	0.442	
Residuals	7	0.08985	0.01284			

Table S7B: Pairwise comparison of microbiota density across samples during mid-season under elevated ozone. Significance levels for each treatment combination are indicated by *p < 0.05; **p < 0.01; ***p < 0.001.

Comparisons	diff	lwr	upr	*p* adjusted	Significance
Control resistant cultivar vs. Control susceptible cultivar	-0.10391667	-0.33077446	0.12294113	0.4770532	
Inoculated susceptible vs. Control susceptible plant	0.20833333	-0.01852446	0.43519113	0.0710621	
Inoculated resistant cultivar vs. Control susceptible cultivar	-0.06341667	-0.29027446	0.16344113	0.7929435	
Inoculated susceptible cultivar vs. Control resistant cultivar	0.31225	0.10934222	0.51515778	0.0059349	**
Inoculated resistant cultivar vs. Control resistant cultivar	0.0405	-0.16240778	0.24340778	0.908505	
Inoculated resistant cultivar vs. Inoculated susceptible cultivar	-0.27175	-0.47465778	-0.06884222	0.012501	*

Table S7C: Permutational multivariant analysis of variance (PERMANOVA) microbiota density across samples during mid-season and ambient environment based on Bray–Curtis dissimilarities in Control samples. Significance levels for each treatment combination are indicated by *p < 0.05; **p < 0.01; ***p < 0.001.

Variable	Df	Sum of sq	R2	F	Pr(>F)	Significance
Treatments	3	0.03403	0.011345	2.976	0.106	
Residuals	7	0.02668	0.003812			

Table S7D: Pairwise comparison of microbiota density across samples during the end of the season and elevated ozone. Significance levels for each treatment combination are indicated by *p < 0.05; **p < 0.01; ***p < 0.001.

Comparisons	diff	lwr	upr	*p* adjusted	Significance
Control resistant cultivar vs. Control susceptible cultivar	0.01929167	-0.250525082	0.2891084	0.9954232	
Inoculated susceptible cultivar vs. Control susceptible cultivar	0.29525	0.025433252	0.5650667	0.0327813	*
Inoculated resistant cultivar vs. Control susceptible cultivar	0.15066667	-0.119150082	0.4204834	0.3446105	
Inoculated susceptible cultivar vs. Control resistant cultivar	0.27595833	0.006141585	0.5457751	0.0451318	*
Inoculated resistant cultivar vs. Control resistant cultivar	0.131375	-0.138441748	0.4011917	0.4499963	
Inoculated resistant cultivar vs. Inoculated susceptible cultivar	-0.14458333	-0.414400082	0.1252334	0.3758588	

Table S7E: Permutational multivariant analysis of variance (PERMANOVA) on the total absolute abundance of microbial communities based on Bray–Curtis dissimilarities in Control samples. Significance levels for each treatment combination are indicated by *p < 0.05; **p < 0.01; ***p < 0.001.

Variable	Df	Sum of sq	R2	F	Pr(>F)	Significance
Treatments	7	0.1481	0.02116	1.881	0.154	
Residuals	13	0.1462	0.01125			

Table S7F: Pairwise comparison of the total absolute abundance of microbial communities in inoculated samples (only the significant treatment combinations are shown in the table). Significance levels for each treatment combination are indicated by *p < 0.05; **p < 0.01; ***p < 0.001.

**Comparisons**	**diff**	**lwr**	**upr**	***p* adjusted**	**Significance**
Ambient environment, mid-season, and resistant cultivar vs. Ambient environment, end of the season, and susceptible cultivar	-0.24672431	-0.43374918	-0.05969943	0.0059222	**
Elevated ozone, mid-season, and resistant cultivar vs. Ambient environment, end of the season, and susceptible cultivar	-0.31988045	-0.50690532	-0.13285558	0.0004468	***
Elevated ozone, mid-season, and resistant cultivar vs. Ambient environment, end of the season, and resistant cultivar	-0.19830876	-0.38533363	-0.01128389	0.0336884	*
Elevated ozone, mid-season, and resistant cultivar vs. Ambient environment, mid-season, and susceptible cultivar	-0.22308397	-0.41010884	-0.0360591	0.0139018	*
Elevated ozone, end of the season, and susceptible cultivar vs. Ambient environment, mid-season, and resistant cultivar	0.26977115	0.08274628	0.45679602	0.0025868	**
Elevated ozone, mid-season, and susceptible cultivar vs. Ambient environment, mid-season, and resistant cultivar	0.21757628	0.03055141	0.40460115	0.0169484	*
Elevated ozone, mid-season, and resistant cultivar vs. Elevated ozone, end of the season, and susceptible cultivar	-0.34292729	-0.52995217	-0.15590242	0.0002051	***
Elevated ozone, mid-season, and resistant cultivar vs. Elevated ozone, mid-season, and susceptible cultivar	-0.29073242	-0.4777573	-0.10370755	0.0012295	**

Table S8: Relative abundance of top 18 bacterial genera across the samples based on Kraken2 and Bracken

Sample id	Cultivar	Inoculation status	Time of sampling	Environment	Relative abundance of top 10 bacterial genera									
					**Xanthomonas**	**Pseudomonas**	**Pantoea**	**Methylobacterium**	**Sphingomonas**	**Methylorubrum**	**Stenotrophomonas**	**Microbacterium**	**Leclercia**	**Brevundimonas**
1XM	Resistant	Inoculated	Mid-season	Ambient	0.021	0.142	0.166	0.252	0.011	0.056	0.003	0.079	0.005	0.008
1XE	Resistant	Inoculated	End of the season	Ambient	0.632	0.131	0.051	0.057	0.044	0.019	0.010	0.002	0.000	0.014
2XM	Resistant	Inoculated	Mid-season	Elevated ozone	0.166	0.027	0.513	0.056	0.008	0.009	0.005	0.036	0.011	0.009
2XE	Resistant	Inoculated	End of the season	Elevated ozone	0.583	0.172	0.021	0.065	0.034	0.028	0.006	0.006	0.015	0.011
3XM	Resistant	Inoculated	Mid-season	Elevated ozone	0.017	0.194	0.117	0.105	0.023	0.027	0.105	0.086	0.055	0.031
3XE	Resistant	Inoculated	End of the season	Elevated ozone	0.871	0.047	0.011	0.012	0.020	0.003	0.009	0.001	0.001	0.002
4XM	Resistant	Inoculated	Mid-season	Ambient	0.049	0.100	0.053	0.119	0.033	0.043	0.010	0.134	0.024	0.032
4XE	Resistant	Inoculated	End of the season	Ambient	0.831	0.076	0.009	0.018	0.016	0.007	0.003	0.000	0.000	0.004
5XM	Resistant	Inoculated	Mid-season	Ambient	0.153	0.195	0.054	0.264	0.015	0.080	0.007	0.036	0.005	0.022
5XE	Resistant	Inoculated	End of the season	Ambient	0.705	0.078	0.020	0.081	0.051	0.032	0.007	0.001	0.000	0.006
6XM	Resistant	Inoculated	Mid-season	Elevated ozone	0.067	0.089	0.229	0.183	0.034	0.056	0.004	0.102	0.026	0.019
6XE	Resistant	Inoculated	End of the season	Elevated ozone	0.331	0.276	0.032	0.107	0.054	0.018	0.002	0.011	0.080	0.010
7XM	Resistant	Control	Mid-season	Elevated ozone	0.002	0.159	0.177	0.152	0.047	0.024	0.037	0.052	0.004	0.061
7XE	Resistant	Control	End of the season	Elevated ozone	0.001	0.396	0.174	0.085	0.114	0.020	0.002	0.005	0.006	0.015
8XM	Resistant	Control	Mid-season	Ambient	0.003	0.165	0.089	0.227	0.025	0.073	0.013	0.073	0.003	0.053
8XE	Resistant	Control	End of the season	Ambient	0.012	0.338	0.090	0.326	0.087	0.074	0.003	0.009	0.001	0.018
9XM	Resistant	Control	Mid-season	Ambient	0.002	0.073	0.019	0.096	0.048	0.013	0.555	0.015	0.008	0.026
9XE	Resistant	Control	End of the season	Ambient	0.002	0.157	0.074	0.351	0.113	0.123	0.025	0.011	0.001	0.024
10XM	Resistant	Control	Mid-season	Elevated ozone	0.098	0.348	0.153	0.068	0.057	0.008	0.050	0.027	0.010	0.016
10XE	Resistant	Control	End of the season	Elevated ozone	0.159	0.295	0.231	0.096	0.143	0.007	0.009	0.004	0.000	0.012
11XM	Resistant	Control	Mid-season	Elevated ozone	0.004	0.339	0.088	0.230	0.016	0.008	0.002	0.035	0.006	0.009
11XE	Resistant	Control	End of the season	Elevated ozone	0.007	0.284	0.434	0.121	0.049	0.016	0.006	0.001	0.000	0.004
12XM	Resistant	Control	Mid-season	Ambient	0.006	0.060	0.056	0.409	0.026	0.051	0.047	0.028	0.005	0.062
12XE	Resistant	Control	End of the season	Ambient	0.004	0.406	0.046	0.218	0.063	0.127	0.027	0.008	0.001	0.030
OXC	Resistant	Control	Base	Green house	0.004	0.005	0.002	0.001	0.001	0.000	0.002	0.000	0.000	0.000
OXI	Resistant	Inoculated	Base	Green house	0.136	0.046	0.019	0.019	0.009	0.001	0.005	0.001	0.000	0.003
1EM	Susceptible	Inoculated	Mid-season	Ambient	0.878	0.024	0.004	0.012	0.002	0.005	0.002	0.001	0.000	0.007
1EE	Susceptible	Inoculated	End of the season	Ambient	0.978	0.013	0.000	0.001	0.000	0.000	0.000	0.018	0.079	0.010
2EM	Susceptible	Inoculated	Mid-season	Elevated ozone	0.893	0.011	0.003	0.010	0.001	0.005	0.001	0.001	0.021	0.005
2EE	Susceptible	Inoculated	End of the season	Elevated ozone	0.940	0.038	0.002	0.000	0.000	0.000	0.005	0.020	0.005	0.006
3EM	Susceptible	Inoculated	Mid-season	Elevated ozone	0.836	0.009	0.105	0.009	0.001	0.004	0.001	0.053	0.000	0.004
3EE	Susceptible	Inoculated	End of the season	Elevated ozone	0.955	0.027	0.001	0.000	0.001	0.000	0.001	0.067	0.003	0.004
4EM	Susceptible	Inoculated	Mid-season	Ambient	0.861	0.048	0.008	0.023	0.002	0.010	0.004	0.004	0.000	0.002
4EE	Susceptible	Inoculated	End of the season	Ambient	0.966	0.024	0.001	0.001	0.001	0.000	0.001	0.023	0.001	0.003
5EM	Susceptible	Inoculated	Mid-season	Ambient	0.796	0.027	0.010	0.037	0.001	0.017	0.005	0.013	0.002	0.002
5EE	Susceptible	Inoculated	End of the season	Ambient	0.949	0.029	0.001	0.001	0.000	0.000	0.002	0.044	0.004	0.002
6EM	Susceptible	Inoculated	Mid-season	Elevated ozone	0.911	0.012	0.007	0.023	0.001	0.010	0.002	0.000	0.001	0.001
6EE	Susceptible	Inoculated	End of the season	Elevated ozone	0.965	0.022	0.000	0.000	0.000	0.000	0.001	0.018	0.000	0.002
7EE	Susceptible	Control	End of the season	Elevated ozone	0.101	0.602	0.137	0.035	0.035	0.022	0.008	0.002	0.000	0.001
8EE	Susceptible	Control	End of the season	Ambient	0.319	0.170	0.199	0.088	0.047	0.020	0.033	0.001	0.000	0.000
9EM	Susceptible	Control	Mid-season	Ambient	0.004	0.091	0.032	0.481	0.043	0.093	0.029	0.002	0.001	0.000
9EE	Susceptible	Control	End of the season	Ambient	0.032	0.501	0.288	0.047	0.031	0.007	0.019	0.001	0.000	0.000
10EM	Susceptible	Control	Mid-season	Elevated ozone	0.043	0.054	0.047	0.091	0.036	0.010	0.006	0.008	0.003	0.033
10EE	Susceptible	Control	End of the season	Elevated ozone	0.125	0.520	0.091	0.047	0.078	0.005	0.005	0.069	0.005	0.046
11EM	Susceptible	Control	Mid-season	Ambient	0.129	0.261	0.083	0.205	0.026	0.010	0.006	0.035	0.001	0.016
11EE	Susceptible	Control	End of the season	Ambient	0.001	0.317	0.072	0.213	0.154	0.063	0.060	0.003	0.063	0.016
12EM	Susceptible	Control	Mid-season	Ambient	0.033	0.104	0.061	0.277	0.032	0.082	0.026	0.018	0.000	0.016
OEC	Susceptible	Control	Base	Green house	0.006	0.017	0.094	0.001	0.001	0.000	0.006	0.003	0.000	0.000
OEI	Susceptible	Inoculated	Base	Green house	0.027	0.030	0.005	0.003	0.005	0.000	0.025	0.000	0.000	0.000

Table S9: Normalized RPKS (Reads Per Kilobase of Sequence) value of eukaryotes genera across samples obtained from the EukDetect pipeline.

Sample id	Cultivar	Inoculation status	Time of sampling	Environment	Top 10 eukaryotic genera									
					Pseudogymnoascus	Zasmidium	Cladosporium	Aureobasidium	Epicoccum	Protomyces	Meira	Moesziomyces	Bullera	Papiliotrema
1EM	Susceptible	Inoculated	Mid-season	Ambient	0.00	0.03	0.01	0.00	0.00	0.36	0.12	2.70	0.04	0.00
1EE	Susceptible	Inoculated	End of the season	Ambient	0.00	0.00	0.00	0.00	0.01	0.00	0.00	0.67	0.00	0.00
1XM	Resistant	Inoculated	Mid-season	Ambient	0.00	0.72	0.19	0.59	0.06	0.60	0.40	1.54	0.15	0.36
1XE	Resistant	Inoculated	End of the season	Ambient	0.00	0.17	0.34	0.55	1.78	0.60	0.00	1.60	1.81	0.55
2EM	Susceptible	Inoculated	Mid-season	Elevated ozone	0.00	0.04	0.06	0.00	0.03	0.74	0.03	8.19	0.23	0.00
2EE	Susceptible	Inoculated	End of the season	Elevated ozone	0.00	0.00	0.00	0.00	0.08	0.02	0.00	0.69	0.07	0.00
2XM	Resistant	Inoculated	Mid-season	Elevated ozone	0.00	0.49	0.83	0.86	0.35	5.58	1.37	36.07	2.17	0.78
2XE	Resistant	Inoculated	End of the season	Elevated ozone	0.00	0.43	1.20	0.65	5.61	0.95	0.05	5.30	2.74	0.94
3EM	Susceptible	Inoculated	Mid-season	Elevated ozone	0.00	0.00	0.03	0.34	0.00	0.52	0.00	2.73	0.09	0.00
3EE	Susceptible	Inoculated	End of the season	Elevated ozone	0.00	0.00	0.00	0.00	0.02	0.00	0.00	0.00	0.00	0.00
3XM	Resistant	Inoculated	Mid-season	Elevated ozone	0.00	0.34	0.56	0.57	0.27	4.84	0.37	11.04	1.29	0.46
3XE	Resistant	Inoculated	End of the season	Elevated ozone	0.00	0.18	0.65	0.45	2.57	0.50	0.02	0.48	4.09	0.37
4EM	Susceptible	Inoculated	Mid-season	Ambient	0.00	0.00	0.00	0.00	0.01	0.39	0.01	1.48	0.04	0.00
4EE	Susceptible	Inoculated	End of the season	Ambient	0.00	0.00	0.00	0.00	0.04	0.04	0.00	0.76	0.04	0.00
4XE	Resistant	Inoculated	End of the season	Ambient	0.00	0.68	0.83	0.60	2.31	0.97	0.10	1.11	2.09	0.48
5EM	Susceptible	Inoculated	Mid-season	Ambient	0.00	0.03	0.00	0.00	0.02	0.35	0.03	1.49	0.08	0.00
5EE	Susceptible	Inoculated	End of the season	Ambient	0.00	0.00	0.00	0.00	0.03	0.03	0.00	0.45	0.10	0.00
5XM	Resistant	Inoculated	Mid-season	Ambient	0.00	0.09	0.18	0.00	0.14	0.36	0.41	1.96	0.12	0.50
5XE	Resistant	Inoculated	End of the season	Ambient	0.00	0.25	0.53	0.00	2.05	1.37	1.64	1.47	2.79	0.53
6EM	Susceptible	Inoculated	Mid-season	Elevated ozone	0.00	0.00	0.00	0.00	0.01	0.06	0.00	2.49	0.04	0.53
6EE	Susceptible	Inoculated	End of the season	Elevated ozone	0.00	0.00	0.00	0.00	0.02	0.00	0.00	0.00	0.05	0.00
6XM	Resistant	Inoculated	Mid-season	Elevated ozone	0.00	0.39	0.65	0.00	0.30	5.24	0.46	22.11	1.00	0.00
6XE	Resistant	Inoculated	End of the season	Elevated ozone	0.00	0.28	1.34	1.67	5.49	1.88	0.00	1.95	4.57	0.74
7EE	Susceptible	Control	End of the season	Elevated ozone	0.00	0.15	0.79	0.53	4.27	0.28	0.00	0.86	4.22	0.39
7XM	Resistant	Control	Mid-season	Elevated ozone	0.00	0.37	0.69	0.46	0.37	2.65	0.27	10.66	1.38	0.66
7XE	Resistant	Control	End of the season	Elevated ozone	0.00	0.12	1.00	0.66	5.34	0.32	0.00	1.22	3.63	0.44
8EE	Susceptible	Control	End of the season	Ambient	0.00	0.10	0.21	0.74	2.16	0.13	0.00	2.89	1.38	0.82
8XM	Resistant	Control	Mid-season	Ambient	0.00	0.41	0.17	0.00	0.55	2.40	0.59	3.42	0.37	0.33
8XE	Resistant	Control	End of the season	Ambient	0.00	0.22	0.28	0.00	1.77	0.24	0.05	3.14	5.29	0.74
9EM	Susceptible	Control	Mid-season	Ambient	0.00	0.36	0.20	0.00	0.11	1.07	0.15	4.55	0.57	0.42
9EE	Susceptible	Control	End of the season	Ambient	0.00	0.04	0.18	0.00	1.19	0.09	0.03	0.92	0.38	0.60
9XM	Resistant	Control	Mid-season	Ambient	0.00	0.50	0.42	0.00	0.34	1.66	0.55	2.77	0.24	0.42
9XE	Resistant	Control	End of the season	Ambient	0.00	0.29	0.47	0.79	2.46	0.79	0.05	2.18	1.71	0.77
10EM	Susceptible	Control	Mid-season	Elevated ozone	0.00	0.96	0.83	0.71	0.56	6.34	0.32	19.23	1.17	0.78
10EE	Susceptible	Control	End of the season	Elevated ozone	0.00	0.01	0.15	0.53	0.30	0.01	0.00	1.17	1.55	0.79
10XM	Resistant	Control	Mid-season	Elevated ozone	0.00	1.17	0.87	1.03	0.68	1.53	12.65	18.08	1.66	0.38
10XE	Resistant	Control	End of the season	Elevated ozone	0.00	0.03	0.31	0.62	0.78	0.08	0.00	1.35	2.29	0.40
11EM	Susceptible	Control	Mid-season	Elevated ozone	0.00	1.77	0.88	0.93	0.83	11.20	0.46	17.09	6.71	0.64
11EE	Susceptible	Control	End of the season	Elevated ozone	0.00	0.57	1.28	1.22	8.07	0.95	0.02	3.14	14.65	2.67
11XM	Resistant	Control	Mid-season	Elevated ozone	0.00	3.17	0.83	0.77	0.70	7.95	0.55	15.08	19.48	0.34
11XE	Resistant	Control	End of the season	Elevated ozone	0.00	0.31	0.69	0.89	4.13	0.29	0.00	2.23	4.82	0.77
12EM	Susceptible	Control	Mid-season	Ambient	0.00	0.28	0.11	0.68	0.15	0.98	0.80	9.53	0.31	0.36
12XM	Resistant	Control	Mid-season	Ambient	0.00	0.30	0.12	0.00	0.20	1.51	0.33	8.85	0.47	0.32
12XE	Resistant	Control	End of the season	Ambient	0.00	0.11	0.15	0.37	0.84	0.13	0.00	4.78	1.79	0.61
OEC	Susceptible	Control	Base	Green house	5.13	0.00	0.00	0.00	0.19	0.00	0.00	0.00	0.00	0.00
OEI	Susceptible	Inoculated	Base	Green house	0.08	0.00	0.00	0.00	0.00	0.00	0.00	0.00	0.00	0.00
OXC	Resistant	Control	Base	Green house	3.40	0.00	2.67	0.00	0.31	0.00	0.00	0.00	0.00	0.00
OXI	Resistant	Inoculated	Base	Green house	0.00	1.01	0.00	0.00	0.00	0.07	0.00	0.98	0.00	0.00

Table S10A: Permutational multivariant analysis of variance (PERMANOVA) of microbial pathways based on Bray–Curtis dissimilarities. Significance levels for each treatment combination are indicated by *p < 0.05; **p < 0.01; ***p < 0.001.

Variable	Df	Sum of sq	Mean Sqs.	F.Model	R2	Pr(>F)	Significance
Environment	1	0.0215	0.021499	1.3167	0.02512	0.2584	
Cultivar	1	0.00262	0.002619	0.1604	0.00306	0.7028	
Inoculation	1	0.0019	0.001898	0.1163	0.00222	0.7612	
Time of sampling	1	0.01013	0.01013	0.6204	0.01184	0.4332	
Environment:Cultivar	1	0.00129	0.001292	0.0791	0.00151	0.814	
Environment:Inoculation status	1	0.00161	0.001607	0.0984	0.00188	0.7756	
Cultivar:Inoculation status	1	0.02972	0.029721	1.8202	0.03473	0.1764	
Environment:Time of sampling	1	0.0315	0.031496	1.9289	0.03681	0.1756	
Cultivar:Time of sampling	1	0.00811	0.008114	0.4969	0.00948	0.4892	
Innoculation status:Time of sampling	1	0.00137	0.001366	0.0837	0.0016	0.8046	
Environment:Cultivar:Inoculation status	1	0.00115	0.001146	0.0702	0.00134	0.8326	
Environment:Cultivar:Time of sampling	1	0.00398	0.00398	0.2438	0.00465	0.6352	
Environment:Inoculation status:Time of sampling	1	0.01283	0.012834	0.786	0.015	0.3814	
Cultivar:Inoculation status:Time of sampling	1	0.20899	0.208991	12.7991	0.24422	0.0014	**
Environment:Cultivar:Inoculation status:Time of sampling	1	0.04551	0.045513	2.7873	0.05319	0.104	
Residuals	29	0.47353	0.016329		0.55336		
Total	44	0.85574			1		

Table S10B: Permutational multivariant analysis of variance (PERMANOVA) of microbial genes based on Bray–Curtis dissimilarities. Significance levels for each treatment combination are indicated by *p < 0.05; **p < 0.01; ***p < 0.001.

Variable	Df	Sum Of Sqs	R2	F	Pr(>F)	Significance
Environment	1	0.02586	0.02767	1.1657	0.2854	
Cultivar	1	0.00379	0.00406	0.171	0.7336	
Inoculation	1	0.00401	0.00429	0.1806	0.727	
Time of sampling	1	0.01453	0.01555	0.6552	0.4262	
Environment:Cultivar	1	0.00154	0.00164	0.0696	0.9034	
Environment:Inoculation status	1	0.00295	0.00315	0.1338	0.7818	
Cultivar:Inoculation status	1	0.02723	0.02915	1.241	0.2664	
Environment:Time of sampling	1	0.03592	0.03844	1.6454	0.1956	
Cultivar:Time of sampling	1	0.00812	0.00869	0.3661	0.5712	
Innoculation status:Time of sampling	1	0.00165	0.00176	0.0739	0.895	
Environment:Cultivar:Inoculation status	1	0.00138	0.00147	0.0588	0.9234	
Environment:Cultivar:Time of sampling	1	0.00449	0.0048	0.1976	0.7074	
Environment:Inoculation status:Time of sampling	1	0.01168	0.0125	0.5156	0.4874	
Cultivar:Inoculation status:Time of sampling	1	0.21206	0.22697	11.834	6.00E-04	***
Environment:Cultivar:Inoculation status:Time of sampling	1	0.05069	0.05426	2.8391	0.0954	
Residuals	29	0.51778	0.5542			
Total	44	0.93429	1			

Table S10C: Permutational multivariant analysis of variance (PERMANOVA) of microbial pathways based on Bray–Curtis dissimilarities during mid and end of the season. Significance levels for each treatment combination are indicated by ****p* < 0.05; *****p* < 0.01; ******p* < 0.001.

Variable	Mid-season			End of the season		
	R2	Pr(>F)	Significance	R2	Pr(>F)	Significance
Environment	0.00088	0.9288		0.00034	0.9744	
Cultivar	0.14094	0.1278		0.29088	0.0194	*
Inoculation	0.04592	0.371		0.02146	0.5004	
Environment:Cultivar	0.01013	0.6776		0.00231	0.8574	
Environment:Inoculation status	0.03693	0.431		0.03231	0.3902	
Cultivar:Inoculation status	0.00561	0.7722		0.00888	0.6762	
Environment:Cultivar:Inoculation status	-0.0003	0.9994		0.00038	0.9798	

Table S11A: Comparison of jaccard index (similarity between the sets of most central nodes) of local network centrality measures between groups. Significance levels for each treatment combination are indicated by *p < 0.05; **p < 0.01; ***p < 0.001.

Pairwise comparison between networks												
	Ambient vs. Elevated ozone				Inoculated vs. Control				Control and ambient environment vs. Combined stress			
**Centrality measures**	**Jaccard index**	***p*(<=Jaccard)**	**Significance**	***p*(>=Jaccard)**	**Jaccard index**	***p*(<=Jaccard)**	**Significance**	***p*(>=Jaccard)**	**Jaccard index**	***p*(<=Jaccard)**	**Significance**	***p*(>=Jaccard)**
Degree	0.069	0.000915	***	0.999879	0.056	0.000081	***	0.999991	0.133	0.01223	*	0.996703
Betweenness centrality	0.136	0.002762	**	0.999208	0.163	0.010359	*	0.996387	0.111	0.00059	***	0.999865
Closeness centrality	0.19	0.031715	*	0.986817	0.163	0.010359	*	0.996387	0.163	0.010359	*	0.996387
eigenvector centrality	0.25	0.171435		0.903429	0.25	0.171435		0.903429	0.02	0	***	1
Hub taxa	0.111	0.143068		0.973988	0	0.017342	*	1	0	0.017342	*	1

Table S11B: Microbial hub taxa across treatment groups

Comparison between ambient and elevated ozone		Comparison between control and inoculated		Comparison between control and ambient environment vs. combined stress	
**Ambient**	**Ozone**	**Control**	**Inoculated**	**Ambient**	**Combined**
Atlantibacter	Exiguobacterium	Brevibacterium	Enterococcus	Acinetobacter	Alcaligenes
Exiguobacterium	Leucobacter	Curtobacterium	Lactococcus	Altererythrobacter	Empedobacter
Mycolicibacterium	Marmoricola	Duganella	Mammaliicoccus	Asaia	Escherichia
Pectobacterium	Nocardioides	Hymenobacter	Mycobacterium	Brucella	Humibacter
Rhodococcus	Weissella	Mycolicibacterium	Weissella	Kocuria	Lactococcus
				Sorangium	Serratia
				Sphingobacterium	Weissella
				Urbifossiella	Yersinia

Table S11C: Adjusted Rand index (similarity between clustering) across treatment groups

**Comparisons**	**ARI**	**p-value**
Ambient vs. Elevated ozone	0.027	0.07
Control vs. Inoculated	0.034	0.021
Control and ambient environment vs. Combined stress	0.103	0

Table S11D: Topological features of the taxonomic networks across various treatments

	Ambient vs. Elevated ozone				Control vs. Inoculated				Ambient vs. Combined stress			
**Topological features**	**Ambient**	**Elevated ozone**	**abs.diff.**	***p*-value**	**Control**	**Inoculated**	**abs.diff.**	***p*-value**	**Control and ambient environment**	**Combined stress**	**abs.diff.**	***p*-value**
Number of components	3	1	2	0.181818	1	1	0	1	1	3	2	0.18981
Clustering coefficient	0.125	0.082	0.043	0.345654	0.12	0.169	0.048	0.410589	0.182	0.121	0.061	0.253746
Moduarity	0.561	0.562	0.002	0.976024	0.544	0.601	0.057	0.168831	0.56	0.611	0.051	0.164835
Positive edge percentage	68.156	65.497	2.659	0.681319	59.563	82.258	22.695	0.102897	83.152	73.62	9.533	0.207792
Edge density	0.036	0.035	0.002	0.904096	0.037	0.038	0.001	0.911089	0.037	0.035	0.002	0.481518
Natural connectivity	0.012	0.012	0	0.723277	0.012	0.013	0	0.535465	0.013	0.013	0	0.713287
Vertex connectivity	1	1	0	1	1	1	0	1	1	1	0	1
Edge connectivity	1	1	0	1	1	1	0	1	1	1	0	1
Average dissimilarity	0.988	0.989	0.001	0.539461	0.989	0.988	0.001	0.669331	0.988	0.989	0.001	0.333666
Average path length	2.702	2.956	0.254	0.703297	2.821	2.765	0.056	0.874126	2.859	3.064	0.205	0.355644
